# Near-cognate tRNAs dominate codon decoding times in simulated ribosomes

**DOI:** 10.1093/nar/gkaf1164

**Published:** 2025-11-06

**Authors:** Fabio Hedayioglu, Emma J Hargreaves, Sathishkumar Kurusamy, James E D Thaventhiran, Anne E Willis, C Mark Smales, Tobias von der Haar

**Affiliations:** Kent Fungal Group, School of Natural Sciences, University of Kent, Canterbury CT2 7NJ, United Kingdom; Industrial Biotechnology Centre, School of Natural Sciences, University of Kent, Canterbury CT2 7NJ, United Kingdom; Industrial Biotechnology Centre, School of Natural Sciences, University of Kent, Canterbury CT2 7NJ, United Kingdom; MRC Toxicology Unit, University of Cambridge, Tennis Court Rd, Cambridge CB2 1QR, United Kingdom; Jantomarna Therapeutics Limited, Magma House 16 Davy Court, Castle Mound Way, Rugby CV23 0UZ, United Kingdom; MRC Toxicology Unit, University of Cambridge, Tennis Court Rd, Cambridge CB2 1QR, United Kingdom; Jantomarna Therapeutics Limited, Magma House 16 Davy Court, Castle Mound Way, Rugby CV23 0UZ, United Kingdom; Industrial Biotechnology Centre, School of Natural Sciences, University of Kent, Canterbury CT2 7NJ, United Kingdom; Jantomarna Therapeutics Limited, Magma House 16 Davy Court, Castle Mound Way, Rugby CV23 0UZ, United Kingdom; National Institute for Bioprocessing Research and Training, Foster Avenue, Mount Merrion, Blackrock, Co. Dublin A94×099,Ireland; Kent Fungal Group, School of Natural Sciences, University of Kent, Canterbury CT2 7NJ, United Kingdom; Jantomarna Therapeutics Limited, Magma House 16 Davy Court, Castle Mound Way, Rugby CV23 0UZ, United Kingdom

## Abstract

The codon sequence of messenger RNAs affects ribosome dynamics, translational control, and transcript stability. Here, we describe an advanced computational modelling tool and its application to studying the effect of different tRNA species on the codon decoding process. Using tRNA abundance data for S*accharomyce cerevisiae*, we show that simulated codon decoding times are sensitive to the abundance of near- and non-cognate tRNAs as well as cognate species. Codon decoding times predicted by models that accurately define near-cognate tRNAs and that are parameterized with high-quality tRNA abundance datasets are highly similar to ribosome dwell times determined using experimental ribosome footprinting data, thereby confirming both the importance of near-cognate tRNAs for the codon decoding process and the general accuracy of our modelling tools.

## Graphical Abstract

**Graphical Abstract:**
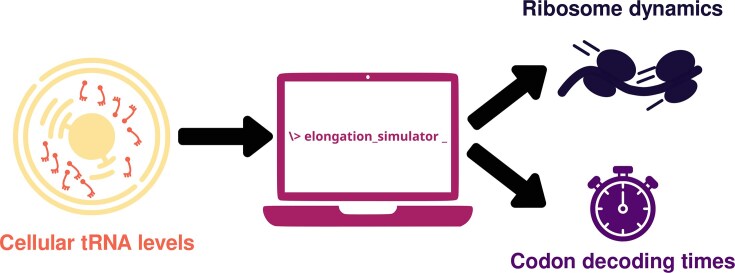

## Introduction

Computational models of codon decoding during protein synthesis are widely used to predict and study gene expression. By enabling the detailed interrogation of specific molecules and their reaction rates, such models have helped elucidate basic features of the protein synthesis machinery in different species [1–4] as well as features of specific translational control pathways [5, 6], have contributed to the study of the evolution of the translational machinery [7–10], and have informed the design of efficient recombinant sequences in biotechnology and medicine [11–14].

During codon decoding transfer RNAs (tRNAs) are randomly sampled from the available cellular tRNA pool. tRNA sampling may deviate from a perfectly mixed system where all pool members are sampled with equal probability, for example, recently used tRNA species may be selected more frequently than expected by chance [15] although it should be noted that evolutionary data do not appear to support this notion [16, 17]. Whether or not tRNA recycling occurs, any deviations from perfectly mixed systems are likely small, and sampling of the tRNA pool is clearly necessary for ribosomes to select cognate tRNAs. The fate of a tRNA that enters the ribosomal A-site is determined by the base pairing pattern between its anticodon and the A-site codon. Depending on the strength of base-pairing, sampled tRNAs may be released from the A-site, or may be accepted and transfer of the nascent peptide onto the amino acid carried by the sampled tRNA may be initiated. If peptidyl transfer occurs, this is followed by translocation of the ribosome onto the next codon, where the sampling process recommences [18, 19].

For each of the 61 sense codons of the canonical genetic code, the cellular tRNA pool can be divided into different tRNA classes depending on the strength of codon: anticodon base pairing. Strongly pairing tRNAs that can initiate peptidyl transfer are designated as “cognates”, in contrast to “non-cognates” which base-pair less strongly or not at all and therefore do not initiate peptidyl transfer. Moreover, available data suggest that tRNAs exist in more complex classes than the simple cognate/non-cognate division. For example, some cognates exclusively form standard Watson:Crick base pairs whereas others form non-standard “wobble” base-pairs. Wobble pairing tRNAs can initiate peptidyl transfer, but in at least some cases do so with lower probability than Watson:Crick pairing tRNAs, and wobble-pairing cognate tRNAs can be rejected from the A-site [20]. Similarly, anticodons of some non-cognate tRNAs do not base pair with the codon at all, whereas others base-pair in one or more of the three nucleotide positions. Where such partial contacts are strong enough to produce non-zero probabilities of initiating peptidyl transfer, the corresponding tRNAs are designated as “near-cognates” [21, 22]. While many studies have addressed the biochemistry and function of cognate tRNAs, both the nature of near-cognates and their role in shaping codon decoding and the genetic code is less well studied. For accurate protein synthesis the reliable acceptance of cognates and rejection of near- and non-cognates are equally important [23], and the relative lack of understanding of near-cognate tRNAs is thus an important knowledge gap.

Codon decoding models need to classify all possible anticodon: codon pairs in terms of the base-pairing patterns described above. Published approaches differ in which classes are considered as explicitly modelled species, with some models only considering the abundance of cognate tRNAs, whereas others consider additional species [24, 25]. Moreover, published modelling approaches also differ in the level of detail with which the codon decoding process is represented. Decoding of individual codons may be modelled as a step with a single rate parameter [26, 27], as two separate codon decoding and translocation steps (2, 28), or as more detailed processes where tRNA accommodation, GTP hydrolysis, peptidyl transfer, and other sub-reactions occur with their own, separate rate parameters [24, 25]. The level of detail with which individual studies represent both tRNA classes and biochemical rate information depends on the study purpose and on the data sources used for parameterization.

In the current study, we extend the general principles we have used previously [1, 25], to develop new modelling software that represents both tRNA classes and biochemical rate information with high levels of detail. Moreover, these models can be parameterized flexibly, allowing users to explore previously inaccessible parameter spaces such as position-dependent changes in reaction parameters. We demonstrate the capabilities of the flexible parameterization approach by exploring in depth how the representation of near cognates as a separate, difficult-to-reject class of tRNAs affects predicted codon decoding times, based on tRNA levels for the model organism *S. cerevisiae*. Finally, we demonstrate that models based on accurate parameters for near-cognate tRNAs result in predicted codon decoding times that are highly similar to experimental ribosome dwell times extracted from ribosome footprinting data.

## Materials and methods

### Model structure

**Figure 1.**
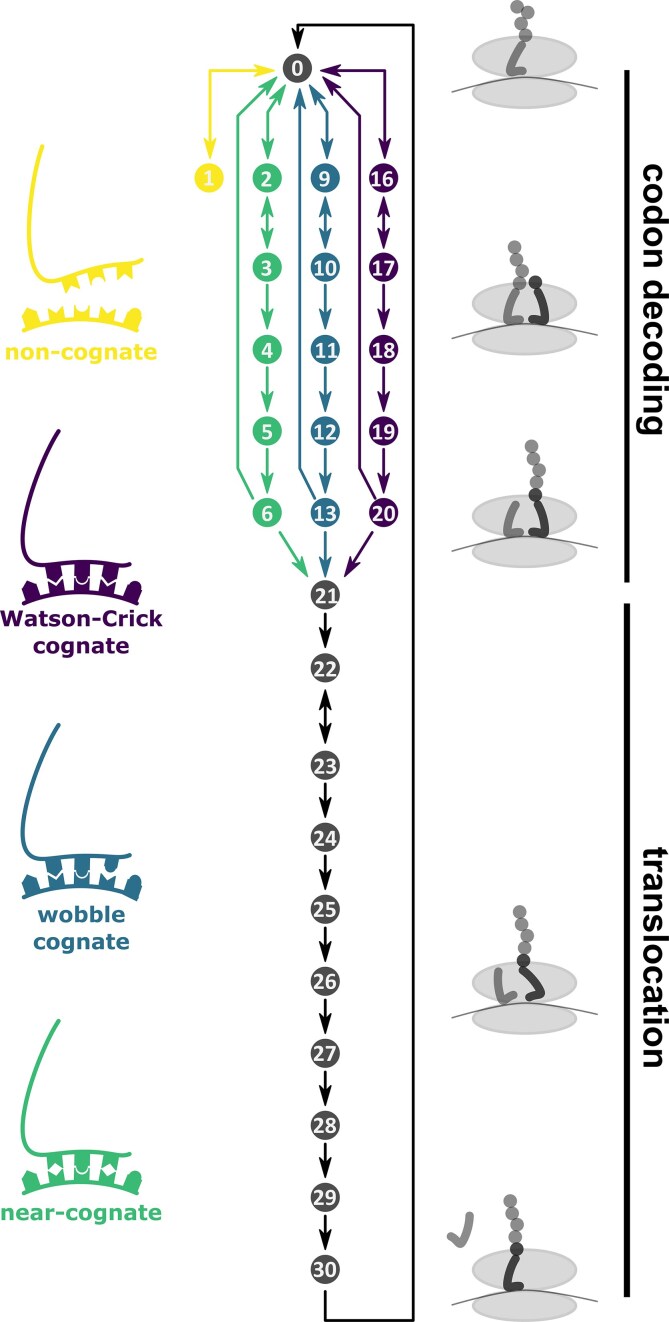
Reactions of the codon decoding cycle. Ribosomes with an empty A-site (state 0) can interact with four distinct classes of tRNAs, where the division of the total cellular tRNA pool into the four classes depends on the nature of the codon located in the A-site. Each ribosome undergoes transient interactions with tRNAs from the pool until aminoacyl-transfer has occurred (state 21), before the eEF2-catalysed translocation process begins which returns the ribosome to state 0 on the subsequent codon.

The new model developed and described herein uses fundamental reaction schemes which we and others have employed previously [1, 12, 24], and considers the four different tRNA classes outlined in the introduction (Watson:Crick pairing cognates, wobble pairing cognates, near-cognates, and non-cognates). The consideration of two separate cognate classes is an extension of our previous model structure. The complete reaction scheme underpinning the model is summarized below (Fig. 1).

Individual tRNAs interact with the ribosome in a sequence of 16 consecutive reaction states that describe the outcomes of the various conformational changes, NTP hydrolysis, aminoacyl-transfer, and translocation reactions that occur during a decoding cycle. The first six states comprise a series of reactions we term codon decoding which culminate in the aminoacyl transfer reaction, whereas the subsequent ten states are termed translocation and include ejection of the P-site tRNA, transfer of the A-site tRNA to the P-site and forward movement of the ribosome by one codon.

Cognate and near-cognate tRNAs can undergo all of the ribosomal reactions, albeit with different rate constants which lead to different reaction rates and in consequence different propensities to reach the aminoacyl-transfer stage (Fig. 1). To allow the modelling software to simulate these different rates efficiently, we define the reactions from initial tRNA binding to peptidyl transfer three times, once for each of the three corresponding tRNA classes. Thus, states 2–6 correspond to the codon decoding phase for Watson–Crick decoding cognates, 7–13 to the codon decoding phase for wobble decoding cognates, and 14–18 to the codon decoding phase for near-cognates. Non-cognates never progress beyond the initial encounter reaction that leads to state 1. For any tRNA which reaches the amino acyl transfer stage rates are then independent of the class of the decoding tRNA, and we only assign a single series of state names to the corresponding reactions (states 21–30).

Since approximations for the rate constants with which the transitions between states occur are known (Supplementary Table S1), we can model this reaction scheme using standard rate constant based approaches including deterministic modelling based on differential equations, or stochastic modelling based on reaction propensities as proposed by Gillespie [29].

### Modelling algorithms

We implemented two separate modelling algorithms, one dedicated to modelling the decoding of individual codons, and a second dedicated to modelling the decoding of messenger RNA (mRNA) sequences. Both were implemented as C++ software and are accessible via the Python classes CodonSimulator and SequenceSimulator. The CodonSimulator class is essentially an implementation of the Gillespie algorithm [29] and allows running stochastic simulations of the decoding of individual codons efficiently.

The SequenceSimulator class relies on a modified implementation of the Gillespie algorithm. A technical issue encountered in modelling the translation of mRNAs containing multiple ribosomes is how to represent the many different reactions that occur during the decoding of a transcript. The decoding of every single codon occurs via the reaction scheme outlined in Fig. 1, so that the total number of distinct reactions related to a sequence of length *N* is N*31 (the number of reactions that can occur on each codon). Moreover, because standard Gillespie algorithms cannot track positional information, this set of reactions needs to be replicated for transcripts decoded by a single ribosome (monosomes), transcripts decoded by two ribosomes simultaneously (disomes), and all N-some variants that can potentially occur on a transcript. Using this approach, it was necessary to implement a system of 968 reactions and 242 components to model a three-codon transcript [3]. While we and others have proposed agent-based approaches that circumvent the need for overly large reaction systems, here we systematically revised the implementation of the algorithm to improve computational performance (Supplementary Fig. S1).

The SequenceSimulator class works with three vectors. One vector contains the codon sequence of the transcript. A second vector contains the current state of each codon of the sequence, which can be either “Available”, “Unavailable” i.e. covered by a ribosome although not located in the ribosomal A-site, or “Decoding” i.e. located in a ribosomal A-site. A third vector contains simulator objects: a simulator object represents an individual ribosome, which is computationally represented as a data structure that contains the current reaction state of the codon, and the reaction parameter set for the A-site codon occupied by that ribosome. There is one set for each codon in “Decoding” state on the sequence, or for each ribosome (an *N*-some is therefore represented by a simulator vector of length *N*).

The information in these three vectors is processed by a simulation engine, which cycles through the following series of steps: (1) All possible reactions in the system are selected, including the possible reactions occurring with elongating ribosomes, but also initiation reactions which add new simulator objects (ribosomes), and termination reactions which remove ribosomes or simulator objects from the vector. Reactions that involve changes in the state vector (i.e. ribosome movement, initiation, or termination) are only allowed if they do not conflict with the location of another ribosome, i.e. ribosomes cannot move onto codons already occupied by other ribosomes. (2) Once the list of possible reactions and their rates has been established, the next reaction and the time required for that reaction are established using the “next reaction” approach [30]. (3) The state and simulator vectors are updated to reflect any changes occurring from the execution of the reaction selected in step 2. This cycle is repeated until a pre-defined stop condition is met, which can be related to elapsed simulation time, computation time, or to a particular number of ribosomal termination events in the modelled system.

Our implementation provides distinct computational advantages, including that the parameters that need to be held in memory at any one time are limited, as well as operating advantages, for example that parameters can be very flexibly configured (this is explored in more detail in the Results section).

**Figure 2.**
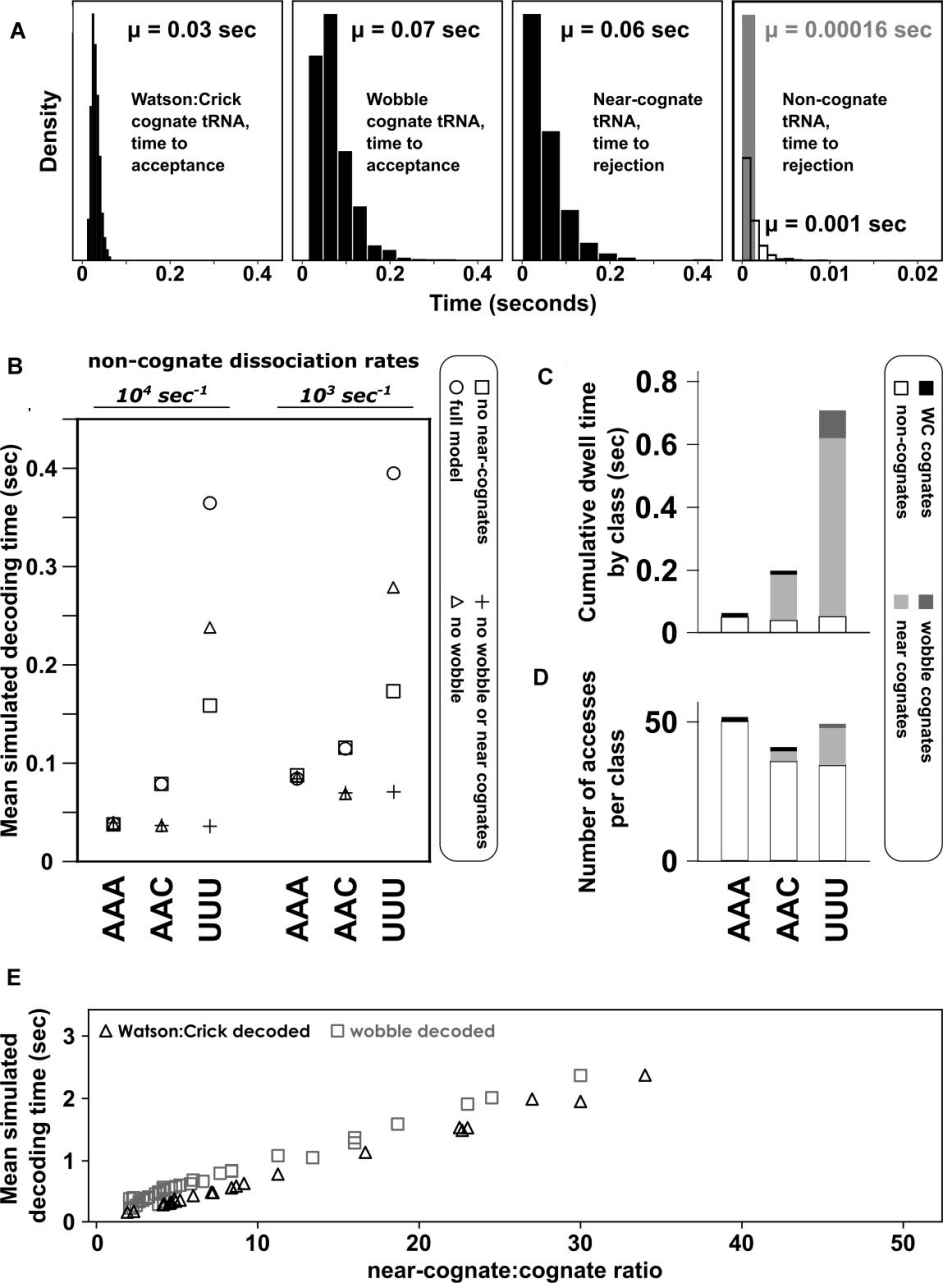
The effect of different model species on codon decoding times. (**A**) Processing times for individual tRNAs in the ribosomal A-site. For non-cognates, two processing times are shown assuming dissociation rate constants of 10^4^ s^−1^ (solid) or 10^3^ s^−1^ (outline). (**B**) Predicted codon decoding times as a function of included species for three representative codons in the baker’s yeast decoding system. “Full model” refers to inclusion of separate Watson:Crick pairing cognate tRNAs, wobble-pairing cognate tRNAs, near-cognate tRNAs, and non-cognate tRNAs. (**C**) The cumulative dwell time of each tRNA class during an average decoding process. Non-cognate dwell times are modelled based on a dissociation rate constant of10^3^ s^−1^. (**D**) Individual tRNA accesses per class in an average codon decoding cycle. For all codons non-cognates constitute the majority of sampled tRNAs, although these are rejected very rapidly and do not contribute strongly to the length of the decoding cycle. (**E**) Dependence of simulated decoding times on near-cognate:cognate ratios.

We further optimized the efficiency of the modelling software through additional measures. Due to the nature of the tRNA-dependent decoding system, the majority of tRNAs in the cellular pool have a “non-cognate” relationship with any given codon. Thus, the majority of tRNA:ribosome interactions are non-cognate binding events, and in fact if all non-cognate interactions are faithfully modelled, the majority of reactions occurring on a modelled transcript are binding and unbinding events of non-cognate tRNAs. Because non-cognates are processed with very rapid rate constants, these events only marginally contribute to the total simulated decoding time (this is explored in detail in the results section and in Fig. 2). Thus, the software has a facility to remove non-cognate interactions from the scheme (non-cognate modelling is disabled by default but can be enabled by the user if desired, Supplementary Fig. S2).

As a second efficiency measure, we implemented a scheme that shortens the time required to simulate transcripts before they reach the steady state, for studies that are predominantly interested in answering questions about the steady state (as is the case for most published studies). If simulation runs are started with empty transcripts, simulations must run until the steady state is reached, and pre-steady state data are typically discarded. To start simulations much nearer to the steady state, we initially calculate the average decoding time for each individual codon, and pre-seed the modelled transcript with the ribosome density expected for a deterministic system. For example, if the initiation rate of the modelled system is 0.1 per second, we distribute ribosomes so that the codons between them take on average 10 s to decode. The resulting polyribosome complex is a system state that has the same ribosome density as the average stochastic state, provided that the effect of ribosome collisions in the system is small (collisions slow down ribosomes and increase ribosome density). The effect of this pre-seeding strategy is length-dependent, for a transcript of 500 codons modelled for 1000 ribosome transits this reduces the required computation time by around 10%–20% (Supplementary Fig. S3).

### Model parameterization

Modelling the reaction scheme outlined in Fig. 1 requires a number of parameterization steps, including (i) establishing lists of known tRNAs to be included in the model as well as determining whether individual tRNA species belong to the cognate, near-cognate, or non-cognate classes, (ii) establishing abundance information for each tRNA species and converting this information into tRNA class abundances (the summed abundance in each of the two cognate, the near-cognate and the non-cognate tRNA classes for each codon), and (iii) establishing rate information for each reaction in the scheme. Note that in the simulations it is assumed that tRNAs are quantitatively charged and in complex with eEF1A:GTP. Conditions of partial aminoacylation, such as amin acid starvation, can however be modelled by reducing tRNA concentrations in the model accordingly.

A summary of the required input parameters is shown in Supplementary Fig. S4. With a complete set of parameters, decoding times for individual codons can be modelled using the CodonSimulator class of the software. If the model is linked to a specific RNA sequence, ribosomal movement on the RNA can additionally be modelled via the SequenceSimulator class.

To provide a flexible tool that allows modelling of as wide a range of scenarios as possible, we provide a set of parameterization tools that allow all model parameters to be freely configured. These are collected under the *set* methods of the simulator objects, including .*setPropensities(), .setInitiationRate(), .setTrnaConcentrations()*, and others.

### Model output

During simulations, the simulator software records all state changes with the times at which they occur in the modelled system. Recording every single reaction produces large amounts of data even though in many contexts it is predominantly the ribosome movement steps that are of interest, and the software therefore provides functionality to switch between coarse-grained state recording (change in ribosome position only) which preserves computing resources, and fine-grained state recording (all state changes in the system).

Both state change events and the time elapsed between them are recorded as variable vectors. In addition to the *event* and *time* vectors produced by both the codon and sequence simulators, the sequence simulator also records all ribosome collisions that occur during the simulation (all events where the distance between two ribosomes becomes zero). In addition, the primary simulation data can be used to calculate derived quantities through *get* methods, including ribosome transit times and individual codon decoding times for each codon.

### Software application

The software is provided as a collection of Python wrappers that access and extend the function of the simulators which are written in C++. Detailed instructions for use are provided via Jupyter Notebooks that exemplify specific use cases, give examples for altering rate constants, near-cognate rules and tRNA abundances and explain how to retrieve results for simulations of longer sequences (https://github.com/tobiasvonderhaar/simulator_manuscript/tree/main/Training). These are provided together with the analysis scripts and data accompanying this manuscript (https://github.com/tobiasvonderhaar/simulator_manuscript).

### Software availability

For use on Windows and Linux systems the Python package(s) containing the modelling software can be downloaded free of charge and installed through the PyPI (https://pypi.org/project/elongation-simulator/) distribution system. Source code is also freely available (https://github.com/fheday/elongation_simulators).

## Results

In addition to offering increased computational efficiency, our new ribosome simulators allow more flexible parameterization compared to existing approaches. Here, we demonstrate the capabilities of this flexible approach with an in-depth analysis of near-cognate tRNAs, a species that strongly shapes the dynamics of codon decoding.

We initially explored the overall effects of inclusion or exclusion of different tRNA species, including near-cognates, on modelled codon decoding times (Fig. 2). In these analyses, rate parameters were taken from biochemical measurements of interactions between ribosomes and different cognate or near-cognate tRNAs [24, 31–34]. Rejection rates on non-cognate tRNAs are estimated between 10^3^ and 10^4^ per second [35, 36], which we use as delimiting values in our initial simulations. A full overview of rate parameters used is shown in Supplementary Table S1.

Individual tRNAs are typically processed by the ribosome within tens of milliseconds (Fig. 2A), where “processing” means either undergoing all reactions up to and including peptidyl transfer (the transitions to state 21 in Fig. 1) or rejection from the A-site (a return to state 0). Non-cognate tRNAs are released within milliseconds or faster, depending on whether dissociation times are assumed to be closer to the upper or lower limits stated above. The total decoding time for a codon is the sum of processing times for all tRNAs that need to be sampled before a tRNA reaches state 21 (the result of a peptidyl transfer reaction). Peptidyl transfer is then followed by ribosomal translocation, before the next tRNA sampling cycle begins.

When different tRNA species are included or excluded from simulations, predicted codon decoding times change for all codons to which these species are relevant. This is illustrated in Fig. 2B for three codons that interact differently with the tRNA pool from Baker’s yeast. In this organism AAA is decoded by the Watson:Crick pairing t(Lys)_UUU_ and according to the near-cognate definition we use, AAA does not have any near-cognate species (A is the nucleotide least able to undergo wobble interactions with unmodified nucleotides). AAC is decoded by the Watson:Crick pairing t(Asn)_GUU_, with t(Tyr)_GUA_ likely acting as a near-cognate. UUU is read by the wobble-pairing t(Phe)_GAA_, with multiple near-cognate species including (t(Ile)_AAU_, t(Ile)_GAU_, t(Leu)_GAG_, and t(Leu)_UAG_. In models based solely on the abundance of cognate tRNAs without distinction between Watson:Crick and wobble-pairing cognates, and where there is no distinction between near- and non-cognates, the decoding process is predicted to occur on very similar time scales for the three illustrative codons (Fig. 2B, crosses). If wobble-decoding tRNAs are modelled with their own specific rate constants [31], the decoding time for UUU (the only wobble-decoded codon in this analysis) is specifically reduced (Fig. 2B, squares). If near-cognates are modelled with their own rate constants [32], AAC and UUU codons are both predicted to be decoded more slowly (Fig. 2B, triangles), and distinguishing all four species leads to the greatest predicted differences in decoding times between the three codons (Fig. 2B, circles). Explicit inclusion of the different tRNA classes in models of the decoding process is thus necessary to capture the full dynamics of this process. Investigation of the dissociation rates of non-cognate tRNAs further reveals that these can be rate-determining under physiological conditions for those codons where competition from near-cognates is limited: thus, simulated decoding times for AAA (no near cognates) are reduced from 0.04 to 0.09 s when non-cognate dissociation rates are decreased from 10^4^ to 10^3^ per second, whereas simulated decoding times for UUU are proportionally much less affected (from 0.37 to 0.4 s).

Interestingly, there is a clear distinction between how many tRNAs of a class need to be rejected during the sampling process, and how long this rejection takes (Fig. 2C and D). For all codons the majority of A-site accesses involves non-cognate tRNAs, whereas the number of near-cognates accessing the A-site is lower (Fig. 2D). However, for codons where near-cognates exist the much slower rejection time means near-cognate rejection is the most time consuming process (Fig. 2C). In consequence, codon decoding times correlate strongly with near-cognate: cognate tRNA ratios, with the specific correlation depending on whether or not codons are decoded by Watson–Crick pairing or cognate tRNAs (Fig. 2E).

### Reaction rate sensitivity in codon decoding

To investigate how different tRNA species affect the dynamics of codon decoding in detail, we conducted a systematic sensitivity analysis for all model parameters. Starting with a basic parameter set as previously published [12], we varied each parameter randomly within a 10% range of the original value and recorded how much codon decoding times for the 61 sense codons varied in response. From the resulting data points, we determined the slope of the decoding times at the original parameter value, which is equivalent to the flux control coefficients used in metabolic control analyses [37, 38].

**Figure 3.**
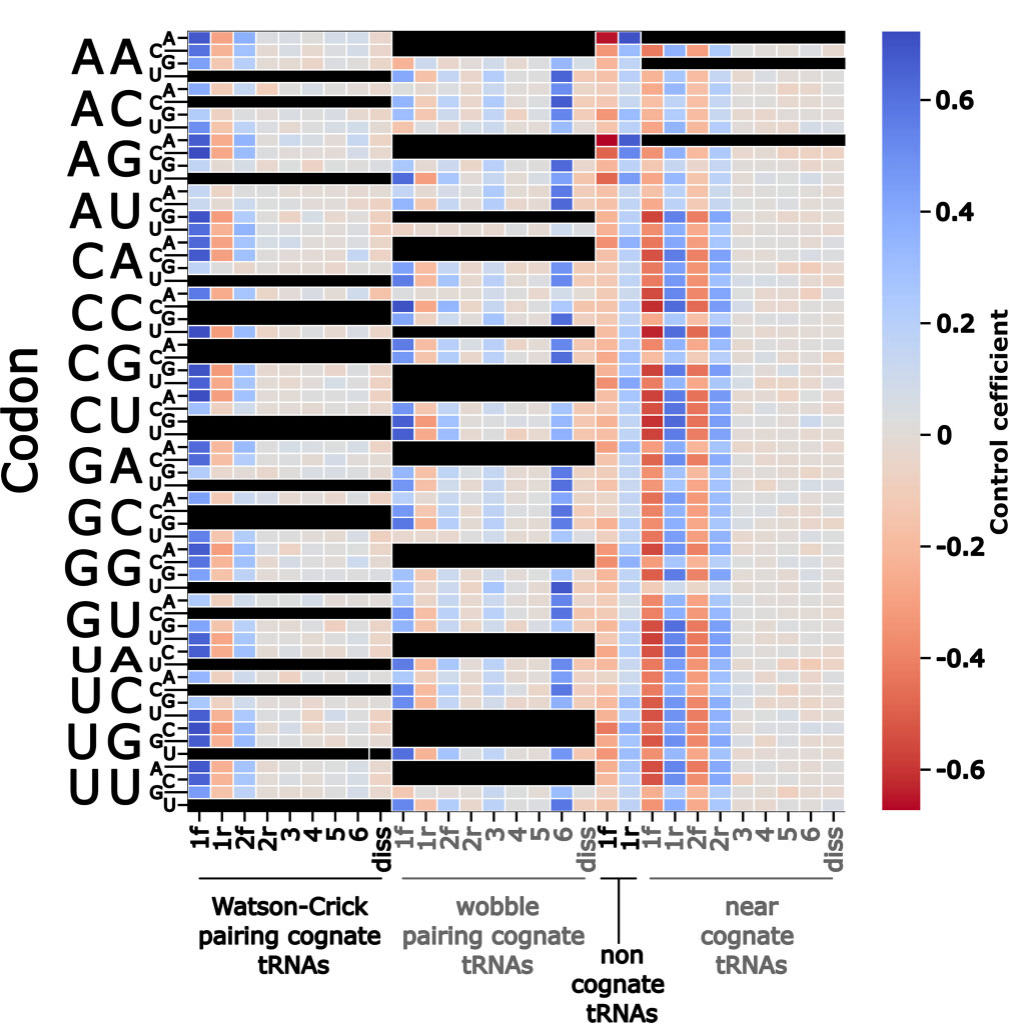
Sensitivity of codon decoding times on individual reaction rates. Colours indicate reaction rates that are correlated (blue), anticorrelated (red), or not correlated (grey) with simulated codon decoding times. tRNA classes which do not exist for individual codons in our model are shown in black.

Figure 3 illustrates how individual ribosomal reactions control codon decoding times, with positive correlation shown in blue, negative correlation in red, and grey indicating low or no correlation. For Watson–Crick pairing cognate tRNAs rate control resides predominantly in the initial formation of the tRNA:ribosome complex, consistent with the view that for strongly pairing cognate tRNAs the concentration (which is proportional to the rate of initial encounter complex formation) drives the efficiency of codon decoding. For wobble-decoding tRNAs rate control is more strongly exerted at later reactions in the decoding scheme, in particular at reaction 6 and at the post-GTP hydrolysis dissociation step. This is consistent with biochemical data showing that wobble decoding cognate tRNAs can be rejected at this step [31], in which case the entire sampling process must be repeated until the next cognate tRNA is encountered. Reducing rejection rates for wobble cognates by either increasing the rate of peptidyl transfer (reaction 6) or by decreasing the rate of dissociation thus reduces the need to undergo the selection process repeatedly, and this controls codon decoding times more strongly than concentrations of wobble-pairing tRNAs. For near-cognate tRNAs, reactions that determine whether these tRNAs progress beyond the initial selection stages most strongly control codon decoding times (reactions 1 and 2 in Fig. 3). Near cognate rejection is particularly slow if these species are rejected post GTP hydrolysis, and more frequent rejection at the initial stages speeds up the rejection process disproportionally. Lastly, for codons where near-cognates exist at low levels or not at all, processing of non-cognates exerts similar levels of rate control as processing of the cognate species. Overall, the strong contributions non- and near-cognate tRNAs make to rate control of the decoding process is consistent with the published finding that ribosomes spend most of their time sorting through ternary complexes [39].

### Defining allowed base pairing patterns in near-cognate tRNAs

Plant *et al.* proposed a working definition for near-cognate tRNAs based on the ability of codon:anticodon pairs to form a Watson:Crick base-pair at the second codon nucleotide, in addition to either Watson:Crick or wobble base-pairs at the other two positions [22]. Models defining near cognates in this way can rank sequences in order of expression levels with high accuracy [12].

Although models based on the Plant *et al.* definition are successful, there is remaining uncertainty regarding the kinds of wobble base-pairs that contribute to distinguishing near-cognate tRNAs from non-cognates. We explored the effect of different wobble base pairs on predicted codon decoding times, by systematically varying allowed base pairing contacts in our decoding model. Allowing or disallowing individual contacts affects which tRNAs are classed as near-cognates for a codon, thereby altering concentrations of its corresponding near-cognate tRNAs.

We initially compiled a comprehensive set of wobble base pairs known to form in solution between all modified bases occurring in yeast tRNA anticodons, and the four natural bases occurring in unmodified mRNA codons (Supplementary Fig. S5). Two of these pairs, G:mcm^5^U and G:U, occur with essential wobble-decoding cognate tRNAs, and cannot be excluded from models without breaking the genetic code. The ten remaining base-pairs can be excluded while still resulting in a functioning genetic code, and we tested comprehensively how the inclusion or exclusion of these base pairs affected model performance. Simulating decoding times with all nucleotide contacts omitted in all possible combinations resulted in >700 datasets, each using differing definitions of near-cognate tRNAs via unique combinations of wobble interactions.

**Figure 4.**
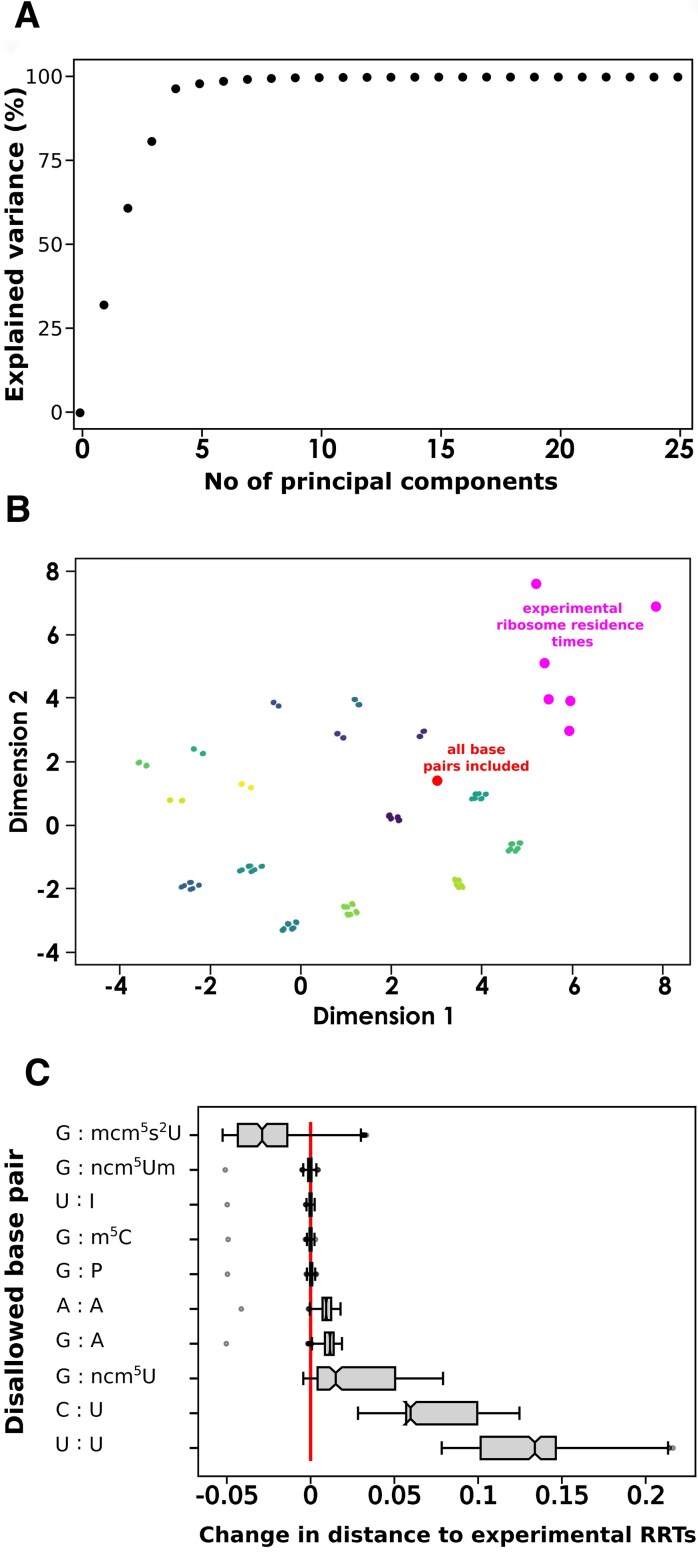
The effect of altered near-cognate definition on modelled codon decoding times. (**A**) Projection of the full, 61-dimensional datasets into reduced numbers of components leads to information loss for small numbers of components, but recovers >99% of information for more than six components. (**B**) Comparison of codon decoding times modelled with different near-cognate definitions and experimentally determined codon decoding times. Experimentally determined codon decoding times are highlighted in magenta, modelled codon decoding times using a near-cognate definition that includes all possible wobble-base pairs is highlighted in red. All other data points are modelled codon decoding times where individual base pairs or combinations of base pairs were removed from the near-cognate definition, and points are coloured to identify different clusters of similar modelling results. (**C**) Removing individual wobble base pairs from the near cognate definition alters the similarity between predicted and experimentally determined codon decoding times. Each box summarizes analyses of 384 data points, with each data point comparing similarity to experimental datasets in the absence or presence of the indicated base pair, alone or in combination with one or more other base pairs.

We initially investigated properties of this dataset by reducing its information content using principal component analysis (PCA) [40]. Each dataset contains information on the decoding times for 61 sense codons. This high dimensionality can be reduced using PCA albeit at the cost of loss of information, for example, projecting information for the 61 codons into two principal components only allows recovering 61% of the available information (Fig. 4A). Interestingly, the first seven components collectively capture >99% of the information of the full, 61-dimensional dataset, implying that the most important underlying structure and patterns within the original high-dimensional data only depend on a subset of the parameters that define that full dataset. One mechanism leading to such a strong reduction in dimensionality could be if only a subset of codons were affected by the changes in near-cognate rules that define each dataset.

We further explored relationships between datasets resulting from different base pairing rules by comparing predicted decoding times to experimentally determined “ribosome residence times” (RRTs). RRTs are estimates of the relative time decoding ribosomes spend on each codon derived from ribosome footprinting data and have been reported by several groups for Baker’s yeast [41–46]. Approaches for recovering RRTs from footprinting data are still debated, and data reported by different groups show variation (Supplementary Fig. S6), but the degree of correlation between different datasets indicates that they contain at least some information on physiological codon decoding times. To compare these experimental datasets to our simulated decoding times we projected distances between the modelled and experimental datasets into 2-dimensional maps, initially exploring a variety of different approaches for dimension reduction and dataset normalization (Supplementary Fig. S7A). This exploratory analysis showed that multidimensional scaling (MDS) [47], when applied to mean normalized datasets, produced well separated clusters, with the number of clusters similar to the number of base pairs considered in the analysis, and with good correlation between mapped and original dataset distances (Supplementary Fig. S7B). We therefore selected this approach for further analysis (Fig. 4B).

In the map (Fig. 4B), different datasets are represented by individual dots, and distances between dots approximate the similarity between corresponding datasets. The modelled decoding times for the full set of base pairs and the experimental datasets are highlighted by red and magenta dots, respectively. The experimental RRT datasets show a spread in this mapping that relates to their quantitative differences. Codon decoding times predicted from simulations in which all candidate wobble base pairs are allowed to contribute to the definition of near-cognate tRNAs (red dot) are outside of the territory occupied by the experimental datasets, indicating that there are systematic differences between the model results and the experimental data.

When individual base pairs, or combinations of base pairs, are removed from the definition of near-cognate tRNAs, concentrations of near-cognates and in consequence modelled codon decoding times change (all points in Fig. 4B other than the highlighted red and magenta points). None of these changes appear to reduce the distance between simulated and experimental data indicating that none of the changes make the simulated data more similar to the experimental datasets, and most changes increased the mapped distance between simulation results and the experimental datasets (Fig. 4B). At least some of the modelled base pairs are thus important to consider in the definition of near-cognate tRNAs, since their omission generates model predictions that become less similar to experimental results.

We validated these findings, and asked which codons produced the strongest effects on simulation results, by analysing what effect omission of individual base pairs had on Euclidean distances between the original, 61-dimensional datasets (Fig. 4C). In this analysis, the relative change in similarity to the experimental datapoints is quantified for omission of each wobble interaction, either in isolation or when paired with all possible combinations of other wobble interactions. Similar to the dimensional mapping (Fig. 4B), this analysis suggests that the predominant effect of removing individual base pairs from the near cognate definition is that simulation results become less similar to experimental results. Only the wobble interaction between guanine and 5-methoxycarbonylmethyl-2-thiouridine (mcm5s2U) produces a small net improvement in the similarity between simulations and experimental data, when omitted from the near-cognate definition scheme. Omission of the uracil:uracil and cytosine:uracil wobble pairs affected the similarity between modelled and experimental data most strongly, indicating that these base pairs contribute most to the definition of near-cognate tRNAs.

These results suggest a working definition for near-cognate tRNAs that includes all base pairs in Supplementary Fig. S5B except for the G:mcm^5^s^2^U base pair (as inclusion of this base pair makes simulated data less similar to experimental data). To provide orthogonal validation of this near-cognate definition, we analysed a published extensive dataset of amino acid misincorporation data [48] to ask whether there is experimental evidence for the amino acid substitutions that would occur under our near cognate rules. We find that of 81 substitutions suggested by our scheme, 59 are observed experimentally (Supplementary Fig. S8). The 22 substitutions for which there is no experimental evidence include two mass neutral substitutions which cannot be detected by mass spectrometry (I < L and L < I), and 10 substitutions with low scores in evolutionary substitution matrices (BLOSUM 62 scores of -2 or -3, Supplementary Fig. S8), which may therefore destabilize proteins meaning they would be less observable in mass spectrometric datasets. Overall, the overlap in predicted and observed amino acid substitutions at least partially corroborates the suggested near-cognate definition.

### Model-based evaluation of tRNA datasets

The quantification of cellular tRNA levels is challenging because the frequent occurrence of modified nucleotides interferes with the reverse transcription step required in most RNA quantification methods. Different approaches have been developed to negate the inhibitory effect of modified nucleotides, including alkaline hydrolysis which releases less modified fragments from full-length tRNAs (Hydro-tRNA-Seq [49]), various strategies for generating full-length cDNAs despite the modifications (QuantMSeq [50], mimSeq [51], and ARM-Seq[52]), and direct RNA sequencing by Nanopore [53]. In Baker’s yeast, tRNA transcription is thought not to be strongly transcriptionally controlled, meaning that in this organism gene copy numbers constitute a useful approximation of tRNA levels [54], and all modelling data discussed so far were generated based on gene copy number counts.

**Figure 5.**
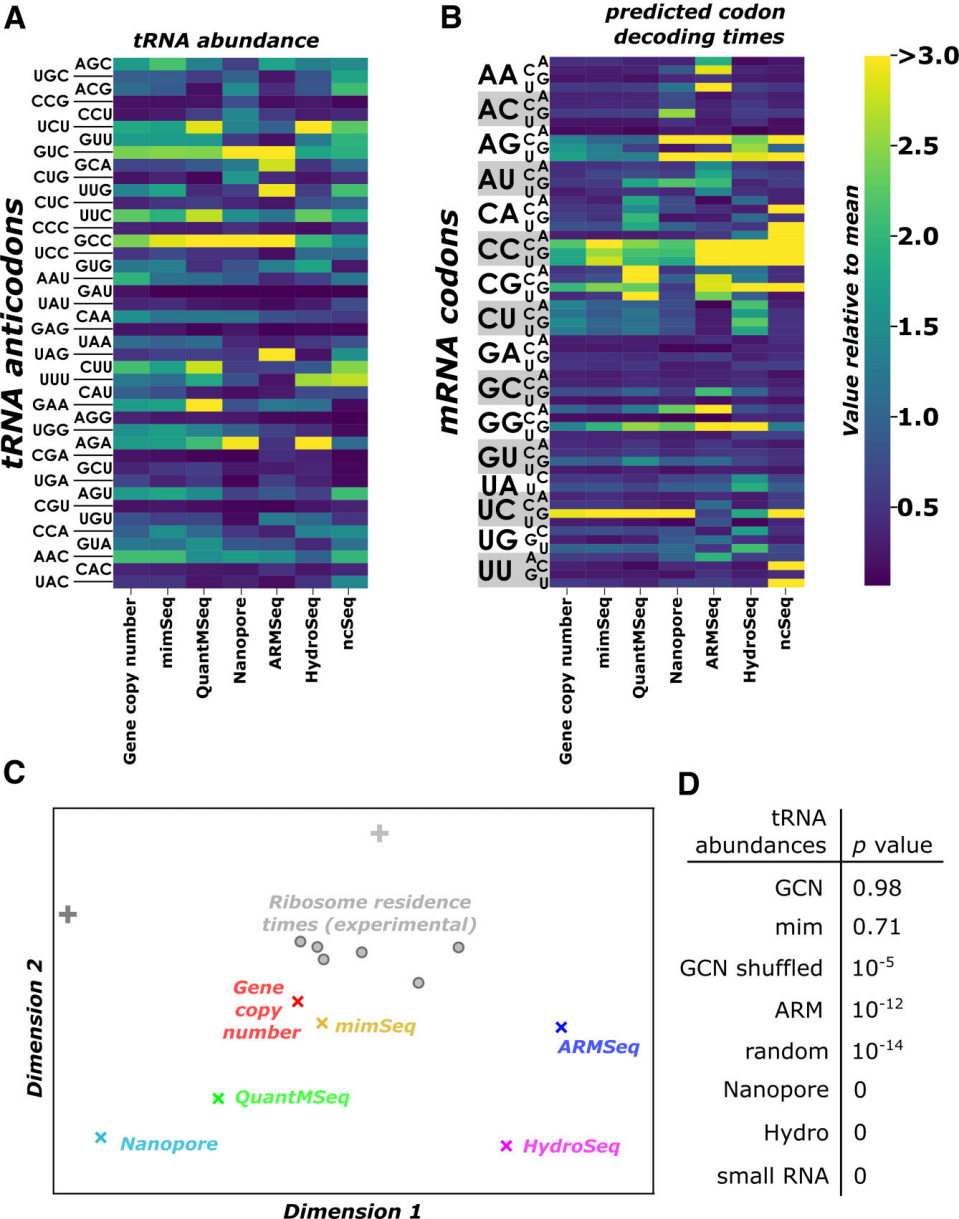
Comparison of model performance based on different tRNA datasets. (**A**) tRNA abundances in datasets generated using different methodologies. (**B**) Codon decoding times simulated using the different tRNA datasets from (A) as input. Data in (A) and (B) are normalized to sample means. (**C**) 2-dimensional mapping of similarities between simulated codon decoding times (coloured “x”), experimentally determined RRTs (grey circles) and control data (“+”). Distances between data reflect similarities between datasets. Control datasets are a randomly drawn set with the same mean and standard deviation as the experimental codon dwell time data (darker “+” symbol), and a randomly re-assigned “GCN” dataset (ligther “+” symbol). (**D**) Statistical comparison of the set of pairwise distances within the experimental data to sets of distances between individual simulated datapoints and the experimental data. *P*-values are based on one-way ANOVA and Tukey’s post-hoc test.

A direct comparison of different tRNA datasets indicates that reported tRNA levels are strongly method-dependent (Fig. 5A, and Supplementary Fig. 9 and Supplementary Table S2). As a consequence, codon decoding times modelled based on the different tRNA levels also differ (Fig. 5B and Supplementary Table S3). We mapped similarities between the different modelled codon decoding times and the experimental RRT datasets discussed earlier, using the same multi-dimensional scaling approach applied above (Fig. 5C). Gene copy number assessment and mimSeq methodologies predicted decoding times that were most similar to the experimental data, with Nanopore, ARMSeq, and HydroSeq approaches producing modelled data that were less similar. In addition, two control datasets were produced by randomly drawing numbers from a normal distribution with the same mean as the experimental abundance data (dark grey cross) and by shuffling the data based on gene copy number simulations (light grey cross). These controls mapped at greater distances from the experimental data sets than the gene copy number and mimSeq simulation data, but at similar or smaller distances compared to the other simulation data. This suggests that simulation data based on gene copy number or mimSeq approaches generate codon decoding times that are more similar to experimental data than expected by chance.

We further investigated the similarity between simulated and experimental decoding time data by comparing pairwise distances between simulated and experimental data sets with pairwise distances only within the experimental datasets. Statistical analysis of these different distance sets resulted in high *P-*values for the gene copy number and mimSeq simulation results (Fig. 5D), indicating that distances between these simulated data and the experimental data are not statistically distinguishable from distances within the experimental data. In contrast, all other simulation data showed low *P-*values in this analysis indicating that their distances to the experimental data differ significantly from distances within the experimental data. This analysis demonstrates that simulation results can generate data of significant similarity to experimental results, provided they are parameterized with accurate tRNA abundance data.

## Discussion

In this study we demonstrate the capabilities of our new, flexibly parameterizable software for simulating ribosome- and tRNA-dependent codon decoding. The reliance on a principal model structure proven in other studies, together with the ability to freely adjust reaction parameters in an amino acid- and sequence-dependent manner, and with the performance advantages offered by an integrated modelling engine, enable easy modelling of scenarios that are not accessible with other, existing modelling software.

We demonstrate the capabilities of the software by exploring how near-cognates, a class of tRNA that has received less attention in past studies compared to cognates, fundamentally affect the dynamics of codon decoding. Introducing a near-cognate tRNA class that is rejected from ribosomal A-sites more slowly than standard non-cognates greatly reduces codon decoding times (Fig. 2B), and for codons for which such tRNAs exist rejecting near-cognates from the ribosomal A-site typically becomes the most time-consuming process (Fig. 2C). These findings are consistent with recent results obtained with independent modelling approaches, which also concluded that sampling ternary complexes is a strongly rate limiting step during codon decoding [39, 55]. By how much the decoding process is slowed depends on the number of near-cognate tRNAs that need to be rejected on average before a cognate is accepted, which is determined by the near-cognate to cognate ratio.

Kothe and Rodnina showed that *E. coli* tRNA(Ala)_UGC_ nearly always progresses to the peptidyl transfer reaction once it reaches the late proof-reading step (the branch point at state 20 in Fig. 1) on Watson–Crick pairing GCA codons, whereas the same tRNA is rejected 40% of the time on cognate but wobble-pairing GCC codons [31]. Rejection of a cognate tRNA doubles the time required for codon decoding under the assumptions of a perfectly mixed system, since the entire sampling process (including slow rejection of any near-cognate tRNAs) needs to be repeated. Given the need to sample for extended times compared to Watson:Crick pairing cognates, high near-cognate:cognate ratios are likely particularly unfavourable for wobble-pairing cognates. In our analyses this penalty for wobble-decoding cognates becomes apparent in the distinct correlations between simulated decoding times and near-cognate ratios for Watson:Crick and wobble-pairing tRNAs in Fig. 2e. This time penalty for wobble-decoders may explain why for amino acids where a single tRNA decodes two possible codons the genetic code shows some of the strongest biases against the wobble-decoded codon. A relevant example are GAA and GAG, both decoded by tRNA(Glu)_UUC_. The wobble-decoded GAG has two abundant near-cognate species, tRNA(Lys)_CUU_ and tRNA(Lys)_UUU_, and is one of most biased-against codons in the yeast genome with an average use ratio of the two glutamic acid inserting codons of 2.4:1 [56].

In addition to asking what overall effects near-cognate tRNAs have in the decoding process, we explored their definition in more detail. Published data suggest that near-cognates are those tRNAs which can undergo partial base pairing between the codon and anticodon [22, 23, 57, 58], and we adopted a working definition requiring formation of a Watson:Crick base pair between the central nucleotides, as well as wobble-base pairs at either or both of the other two positions [22]. Which kinds of wobble-base pairs sufficiently stabilize contacts to make tRNAs behave like near-cognates is still unclear. To explore this question we chose a “brute force” approach in which we allowed or disallowed all possible wobble contacts that can be omitted without breaking the genetic code, in isolation or in combination with all other possible contacts. By comparing the resulting decoding time predictions to experimentally determined RRTs from a number of published studies, we show that disallowing wobble contacts typically made predictions less similar to the experimental data (Fig. 4), consistent with the notion that these contacts contribute to near-cognate behaviour in real tRNAs, and that such near-cognate tRNAs affect codon decoding times. Moreover, very recent data exploring ribosomal pausing in response to depletion or overexpression of individual arginine-inserting tRNAs [58] show good agreement with our modelling results for the rare tRNA(Arg)_CCU_. All codons that experimentally show reduced pausing in response to depletion of this tRNA, or increased pausing in response to its overexpression, are predicted to be near-cognates in our definition. For a second tRNA tested in that study (tRNA(Arg)_UCU_), agreement with our near-cognate definition is less good, with one of three codons showing increased pausing upon t(Arg)_UCU_ overexpression being defined as near-cognate codons for this tRNA in our scheme. This lack of agreement may indicate that near-cognate definitions still needs further refinement in order to capture physiological translation accurately, but we note that overexpression of t(Arg)_UCU_ did not appear to affect pausing on its cognate AGA codon in the experimental study, whereas it did reduce pausing on an unrelated CCG codon, so that it is not clear in how far effects of t(Arg)_UCU_ overexpression are direct results of altered tRNA competition.

The salient parameter that distinguishes near-cognates from non-cognates (and equally wobble-decoding cognates from Watson–Crick decoding ones) is most likely the strength of base pairing between the tRNA anticodon and mRNA codon, which needs to be sufficiently strong to displace a series of “gatekeeper” nucleotides in the small subunit ribosomal RNA (rRNA), thereby inducing conformational changes that are propagated throughout the ribosome [22, 59]. The probability of this displacement correlates with the affinity of the codon:anticodon pair, which explains both why weaker binding (wobbling) cognates can sometimes be wrongly rejected, while stronger binding near-cognates are more slowly and less reliably rejected than weaker non-cognates. Base-pairing strength is a continuous parameter, and we would expect that in reality individual tRNA:codon pairs are located on a continuum ranging from more near-cognate-like to more non-cognate-like, rather than behaving uniformly within two clearly separable classes. We currently lack the biochemical evidence to model this continuum in detail, but it is likely that the development of approaches that modulate the rate constants with which tRNAs interact in the ribosomal A-site based on the codon:anticodon affinity will further improve the accuracy with which codon decoding times can be predicted.

## Supplementary Material

gkaf1164_Supplemental_File

## Data Availability

Analysis scripts and data are available at https:/github.com/tobiasvonderhaar/simulator_manuscript or via doi: 10.5281/zenodo.17099494. Software source code is available at https://github.com/fheday/elongation_simulators. Python software can be installed using the PyPI distribution system (https://pypi.org/project/elongation-simulator/) with the command “pip install elongation-simulator”.

## References

[1] Chu D, Barnes DJ, von der Haar T. The role of tRNA and ribosome competition in coupling the expression of different mRNAs in Saccharomyces cerevisiae. Nucleic Acids Res. 2011;39:6705–14. 10.1093/nar/gkr300. 21558172 PMC3159466

[2] Ciandrini L, Stansfield I, Romano MC. Ribosome traffic on mRNAs maps to gene ontology: genome-wide quantification of translation initiation rates and polysome size regulation. PLoS Comput Biol. 2013;9:e1002866. 10.1371/journal.pcbi.1002866. PMC3561044 23382661

[3] Matsuura T, Tanimura N, Hosoda K et al. Reaction dynamics analysis of a reconstituted *Escherichia coli* protein translation system by computational modeling. Proc Natl Acad Sci USA. 2017;114:E1336–44. 10.1073/pnas.1615351114. 28167777 PMC5338406

[4] Rudorf S . Efficiency of protein synthesis inhibition depends on tRNA and codon compositions. PLoS Comput Biol. 2019;15:e1006979. 10.1371/journal.pcbi.1006979. 31369559 PMC6692046

[5] Bastide A, Peretti D, Knight JRP et al. RTN3 is a novel cold-induced protein and mediates neuroprotective effects of RBM3. Curr Biol. 2017;27:638–50. 10.1016/j.cub.2017.01.047. 28238655 PMC5344685

[6] Malik Y, Kulaberoglu Y, Anver S et al. Disruption of tRNA biogenesis enhances proteostatic resilience, improves later-life health, and promotes longevity. PLoS Biol. 2024;22:e3002853. 10.1371/journal.pbio.3002853. PMC11495624 39436952

[7] dos Reis M . Solving the riddle of codon usage preferences: a test for translational selection. Nucleic Acids Res. 2004;32:5036–44. 10.1093/nar/gkh834. PMC521650 15448185

[8] Zur H, Tuller T. Predictive biophysical modeling and understanding of the dynamics of mRNA translation and its evolution. Nucleic Acids Res. 2016;44:gkw764. 10.1093/nar/gkw764. PMC5100582 27591251

[9] Deng Y, de Lima Hedayioglu F, Kalfon J et al. Hidden patterns of codon usage bias across kingdoms. J R Soc Interface. 2020;17:20190819. 10.1098/rsif.2019.0819. 32070219 PMC7061699

[10] Maheshwari AJ, Calles J, Waterton SK et al. Engineering tRNA abundances for synthetic cellular systems. Nat Commun. 2023;14:4594. 10.1038/s41467-023-40199-9. PMC10390467 37524714

[11] Racle J, Overney J, Hatzimanikatis V. A computational framework for the design of optimal protein synthesis. Biotech Bioeng. 2012;109:2127–33. 10.1002/bit.24463. 22334333

[12] Chu D, Kazana E, Bellanger N et al. Translation elongation can control translation initiation on eukaryotic mRNAs. EMBO J. 2014;33:21–34. 10.1002/embj.201385651. 24357599 PMC3990680

[13] Trösemeier J-H, Rudorf S, Loessner H et al. Optimizing the dynamics of protein expression. Sci Rep. 2019;9:7511. 10.1038/s41598-019-43857-5. 31101858 PMC6525252

[14] Ranaghan MJ, Li JJ, Laprise DM et al. Assessing optimal: inequalities in codon optimization algorithms. BMC Biol. 2021;19:36. 10.1186/s12915-021-00968-8. 33607980 PMC7893858

[15] Cannarozzi G, Schraudolph NN, Faty M et al. A role for codon order in translation dynamics. Cell. 2010;141:355–67. 10.1016/j.cell.2010.02.036. 20403329

[16] Hussmann JA, Press WH. Local correlations in codon preferences do not support a model of tRNA recycling. Cell Rep. 2014;8:1624–9. 10.1016/j.celrep.2014.08.012. 25199837

[17] Novoa EM, Jungreis I, Jaillon O et al. Elucidation of codon usage signatures across the domains of life. Mol Biol Evol. 2019;36:2328–39. 10.1093/molbev/msz124. 31220870 PMC6759073

[18] Wilson DN, Doudna Cate JH. The structure and function of the eukaryotic ribosome. Cold Spring Harb Perspect Biol. 2012;4:a011536–. 10.1101/cshperspect.a011536. 22550233 PMC3331703

[19] Knight JRP, Garland G, Pöyry T et al. Control of translation elongation in health and disease. Dis Model Mech. 2020;13:dmm043208. 10.1242/dmm.043208. 32298235 PMC7104864

[20] Agris PF, Vendeix FAP, Graham WD. tRNA’s wobble decoding of the genome: 40 years of modification. J Mol Biol. 2007;366:1–13. 10.1016/j.jmb.2006.11.046. 17187822

[21] Blanchet S, Cornu D, Hatin I et al. Deciphering the reading of the genetic code by near-cognate tRNA. Proc Natl Acad Sci USA. 2018;115:3018–23. 10.1073/pnas.1715578115. PMC5866558 29507244

[22] Plant EP, Nguyen P, Russ JR et al. Differentiating between near- and non-cognate codons in *Saccharomyces cerevisiae*. PLoS One. 2007;2:e517. 10.1371/journal.pone.0000517. PMC1885216 17565370

[23] Manickam N, Joshi K, Bhatt MJ et al. Effects of tRNA modification on translational accuracy depend on intrinsic codon–anticodon strength. Nucleic Acids Res. 2016;44:1871–81. 10.1093/nar/gkv1506. 26704976 PMC4770228

[24] Fluitt A, Pienaar E, Viljoen H. Ribosome kinetics and aa-tRNA competition determine rate and fidelity of peptide synthesis. Comput Biol Chem. 2007;31:335–46. 10.1016/j.compbiolchem.2007.07.003. PMC2727733 17897886

[25] Chu D, Zabet N, der Haar T. A novel and versatile computational tool to model translation. Bioinformatics. 2012;28:292–3. 10.1093/bioinformatics/btr650. 22113083

[26] Dana A, Tuller T. The effect of tRNA levels on decoding times of mRNA codons. Nucleic Acids Res. 2014;42:9171–81. 10.1093/nar/gku646. PMC4132755 25056313

[27] Shah P, Ding Y, Niemczyk M et al. Rate-limiting steps in yeast protein translation. Cell. 2013;153:1589–601. 10.1016/j.cell.2013.05.049. 23791185 PMC3694300

[28] Gritsenko AA, Hulsman M, Reinders MJT et al. Unbiased quantitative models of protein translation derived from ribosome profiling data. PLoS Comput Biol. 2015;11:e1004336. 10.1371/journal.pcbi.1004336. PMC4537299 26275099

[29] Gillespie DT . A general method for numerically simulating the stochastic time evolution of coupled chemical reactions. J Comput Phys. 1976;22:403–34. 10.1016/0021-9991(76)90041-3.

[30] Gibson MA, Bruck J. Efficient exact stochastic simulation of chemical systems with many species and many channels. J Phys Chem A. 2000;104:1876–89. 10.1021/jp993732q.

[31] Kothe U, Rodnina MV. Codon reading by tRNAAla with modified uridine in the wobble position. Mol Cell. 2007;25:167–74. 10.1016/j.molcel.2006.11.014. 17218280

[32] Mittelstaet J, Konevega AL, Rodnina MV. Distortion of tRNA upon near-cognate codon recognition on the ribosome. J Biol Chem. 2011;286:8158–64. 10.1074/jbc.M110.210021. 21212264 PMC3048702

[33] Gromadski KB, Rodnina MV. Kinetic determinants of high-fidelity tRNA discrimination on the ribosome. Mol Cell. 2004;13:191–200. 10.1016/S1097-2765(04)00005-X. 14759365

[34] Gromadski KB, Daviter T, Rodnina MV. A uniform response to mismatches in codon-anticodon complexes ensures ribosomal fidelity. Mol Cell. 2006;21:369–77. 10.1016/j.molcel.2005.12.018. 16455492

[35] Mustafi M, Weisshaar JC. Simultaneous binding of multiple EF-Tu copies to translating ribosomes in live *Escherichia coli*. mBio. 2018;9:e02143–17. 10.1128/mBio.02143-17. PMC5770553 29339430

[36] Rudorf S, Thommen M, Rodnina MV et al. Deducing the kinetics of protein synthesis *in vivo* from the transition rates measured *in vitro*. PLoS Comput Biol. 2014;10:e1003909. 10.1371/journal.pcbi.1003909. 25358034 PMC4214572

[37] Kacser H, Burns JA. The control of flux. Symp Soc Exp Biol. 1973;27:65–104. 4148886

[38] Heinrich R, Rapoport TA. A linear steady-state treatment of enzymatic chains. general properties, control and effector strength. Eur J Biochem. 1974;42:89–95. 10.1111/j.1432-1033.1974.tb03318.x. 4830198

[39] Moore PB . On the response of elongating ribosomes to forces opposing translocation. Biophys J. 2024;123:3010–23. 10.1016/j.bpj.2024.05.032. 38845199 PMC11427781

[40] Jolliffe IT, Cadima J. Principal component analysis: a review and recent developments. Phil Trans R Soc A. 2016;374:20150202. 10.1098/rsta.2015.0202. PMC4792409 26953178

[41] Gardin J, Yeasmin R, Yurovsky A et al. Measurement of average decoding rates of the 61 sense codons *in vivo*. eLife. 2014;3:e03735. 10.7554/eLife.03735. PMC4371865 25347064

[42] Pop C, Rouskin S, Ingolia NT et al. Causal signals between codon bias, mRNA structure, and the efficiency of translation and elongation. Mol Syst Biol. 2014;10:770. 10.15252/msb.20145524. PMC4300493 25538139

[43] Weinberg DE, Shah P, Eichhorn SW et al. Improved ribosome-footprint and mRNA measurements provide insights into dynamics and regulation of yeast translation. Cell Rep. 2016;14:1787–99. 10.1016/j.celrep.2016.01.043. PMC4767672 26876183

[44] Fang H, Huang Y-F, Radhakrishnan A et al. Scikit-ribo enables accurate estimation and robust modeling of translation dynamics at codon resolution. Cell Syst. 2018;6:180–191.e4. 10.1016/j.cels.2017.12.007. 29361467 PMC5832574

[45] Do Couto Bordignon P, Pechmann S. Inferring translational heterogeneity from *Saccharomyces cerevisiae* ribosome profiling. FEBS J. 2021;288:4541–59. 10.1111/febs.15748. 33539640

[46] Wu CCC, Zinshteyn B, Wehner KA et al. High-resolution ribosome profiling defines discrete ribosome elongation states and translational regulation during cellular stress. Mol Cell. 2019;73:959–970.e5. 10.1016/j.molcel.2018.12.009e5. 30686592 PMC6411040

[47] Kruskal JB . Multidimensional scaling by optimizing goodness of fit to a nonmetric hypothesis. Psychometrika. 1964;29:1–27. 10.1007/BF02289565.

[48] Landerer C, Poehls J, Toth-Petroczy A. Fitness effects of phenotypic mutations at proteome-scale reveal optimality of translation machinery. Mol Biol Evol. 2024;41:msae048. 10.1093/molbev/msae048. 38421032 PMC10939442

[49] Gogakos T, Brown M, Garzia A et al. Characterizing expression and processing of precursor and mature human tRNAs by Hydro-tRNAseq and PAR-CLIP. Cell Rep. 2017;20:1463–75. 10.1016/j.celrep.2017.07.029. 28793268 PMC5564215

[50] Pinkard O, McFarland S, Sweet T et al. Quantitative tRNA-sequencing uncovers metazoan tissue-specific tRNA regulation. Nat Commun. 2020;11:4104. 10.1038/s41467-020-17879-x. 32796835 PMC7428014

[51] Behrens A, Rodschinka G, Nedialkova DD. High-resolution quantitative profiling of tRNA abundance and modification status in eukaryotes by mim-tRNAseq. Mol Cell. 2021;81:1802–1815.e7. 10.1016/j.molcel.2021.01.028e7. PMC8062790 33581077

[52] Cozen AE, Quartley E, Holmes AD et al. ARM-seq: alkB-facilitated RNA methylation sequencing reveals a complex landscape of modified tRNA fragments. Nat Methods. 2015;12:879–84. 10.1038/nmeth.3508. PMC4553111 26237225

[53] Lucas MC, Pryszcz LP, Medina R et al. Quantitative analysis of tRNA abundance and modifications by nanopore RNA sequencing. Nat Biotechnol. 2024;42:72–86. 10.1038/s41587-023-01743-6. 37024678 PMC10791586

[54] Ikemura T . Correlation between the abundance of yeast transfer RNAs and the occurrence of the respective codons in protein genes. J Mol Biol. 1982;158:573–97. 10.1016/0022-2836(82)90250-9. 6750137

[55] Rudorf S, Lipowsky R. Protein synthesis in *E. coli*: dependence of codon-specific elongation on tRNA concentration and codon usage. PLoS One. 2015;10:e0134994. 10.1371/journal.pone.0134994. PMC4535986 26270805

[56] Nakamura Y . Codon usage tabulated from international DNA sequence databases: status for the year 2000. Nucleic Acids Res. 2000;28:292–. 10.1093/nar/28.1.292. 10592250 PMC102460

[57] Kramer EB, Vallabhaneni H, Mayer LM et al. A comprehensive analysis of translational missense errors in the yeast *Saccharomyces cerevisiae*. RNA. 2010;16:1797–808. 10.1261/rna.2201210. 20651030 PMC2924539

[58] Aguilar Rangel M, Stein K, Frydman J. A machine learning approach uncovers principles and determinants of eukaryotic ribosome pausing. Sci Adv. 2024;10:eado0738. 10.1126/sciadv.ado0738. 39423268 PMC11488575

[59] Ogle JM, Murphy FV, Tarry MJ et al. Selection of tRNA by the ribosome requires a transition from an open to a closed form. Cell. 2002;111:721–32. 10.1016/S0092-8674(02)01086-3. 12464183

