# Supplementary material for: Near-cognate tRNAs dominate codon decoding times in simulated ribosomes
Source: Nucleic Acids Res. 2025 Nov 6;53(20):gkaf1164. doi: 10.1093/nar/gkaf1164 (PMC12596189; doi:10.1093/nar/gkaf1164)
Supplement: gkaf1164_Supplemental_File [file gkaf1164_supplemental_file.pdf]

Supplemental data

1) Software performance

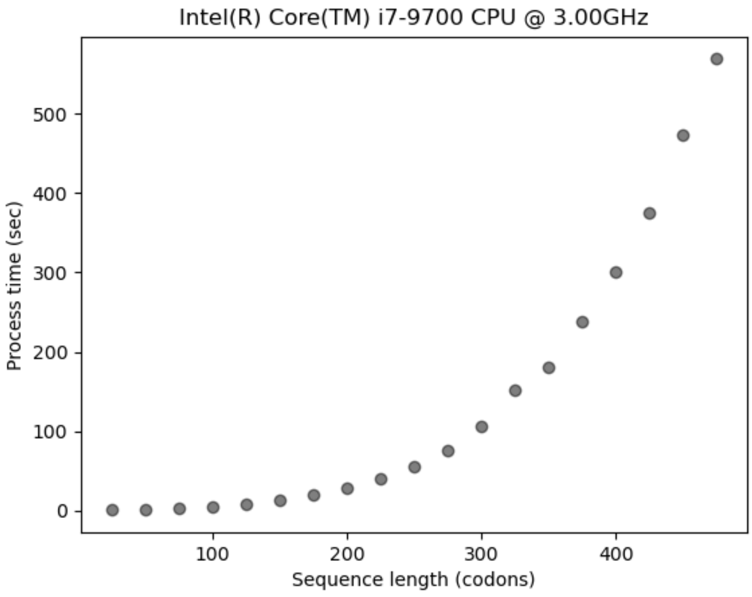

Supplemental figure 1. Modelling performance on a 1.8 GHz Intel i7 8565U CPU. Sequences of increasing length encoding parts of Glutathione-S-Transferase were modelled using a termination criterion of 1000 terminated ribosomes.

2) Inclusion of non-cognate tRNAs

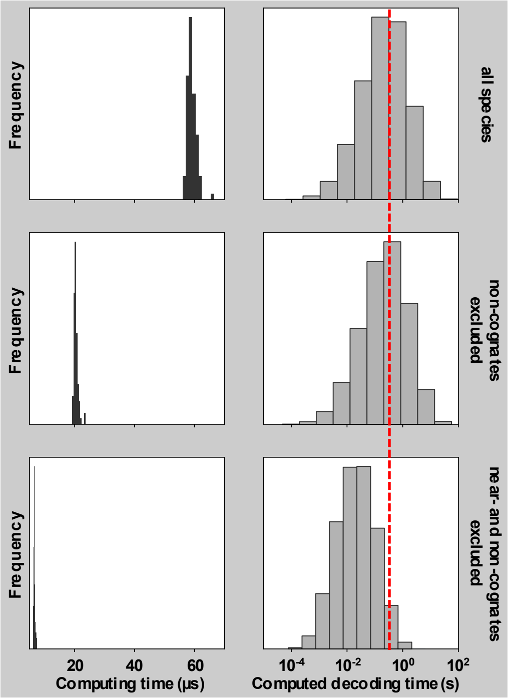

Supplemental figure 2. Faithful modelling of all non-cognate interactions strongly increases computing time without significantly affecting modelling results. The top panel compares the computing time required (left) and the computed decoding time results (right) for a model that includes all species, including all non-cognate tRNAs. Omission of non-cognates reduces the computing time without affecting model results (center), whereas omission of near-cognates affects both computing time and model results (bottom).

3) Prepopulating transcripts

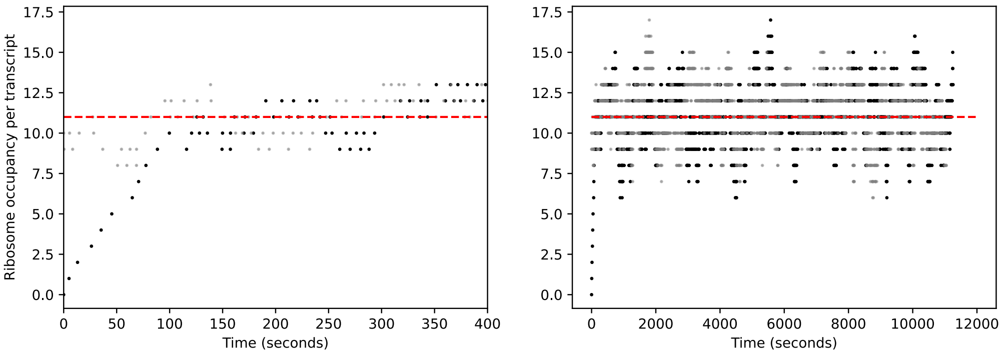

Supplemental figure 3. Ribosome pre-seeding shortens pre-steady state simulation time. Development of pre-steady state ribosome occupancies of model runs that begin with empty transcripts (`sim.setPrepopulate(False)` , black dots), or with transcripts pre-seeded with a ribosome occupancy solution corresponding to the deterministic steady state solution (`sim.setPrepopulate(True)` , grey dots). The panels show identical data from a simulation modelling translation of a yeast codon optimised glutathione-S-transferase gene, focusing on the first 400 seconds (left) or showing the full simulation (right). The red line shows the median ribosome occupancy from in the simulations excluding the first 250 seconds (11 ribosomes per transcripts in simulations both with and without ribosome pre-seeding).

4) Parameter requirements

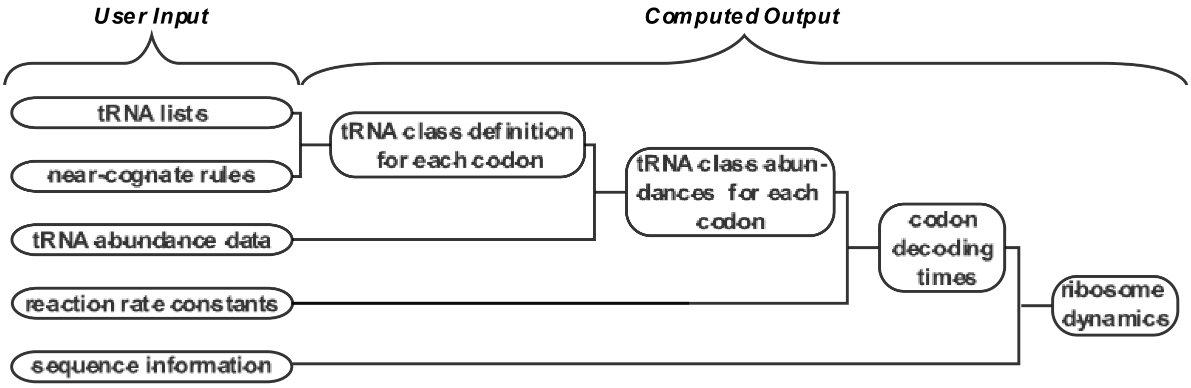

Supplemental figure 4. Parameter requirements of the modelling software.

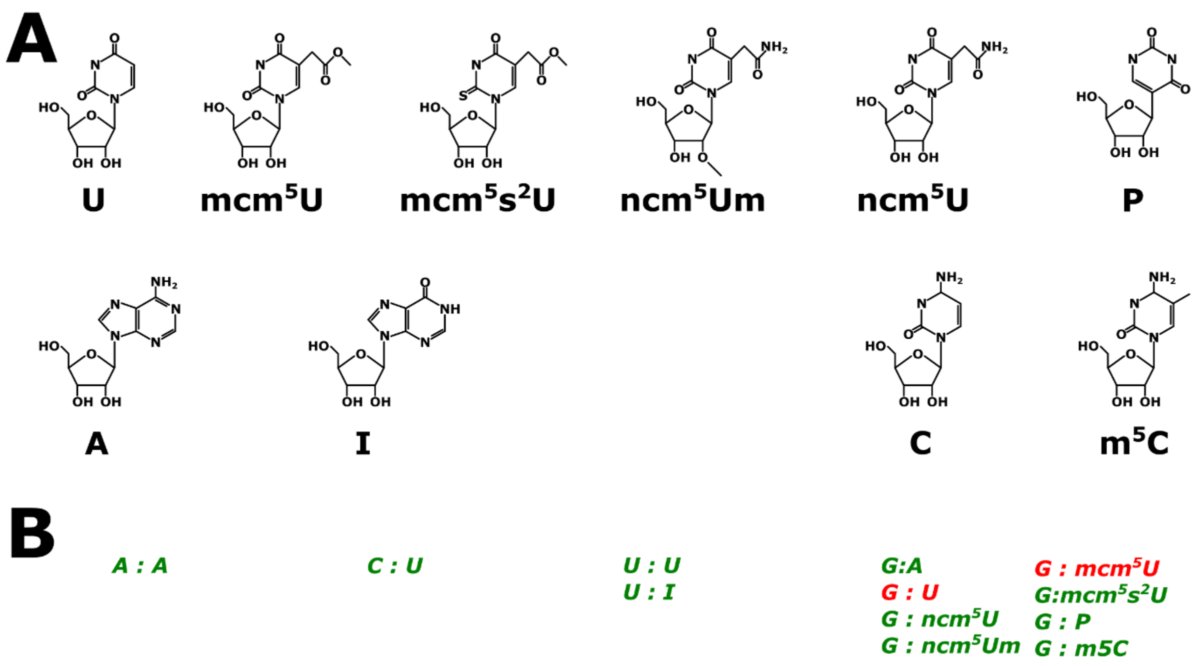

Supplemental Figure 5. Modified nucleotides (A) and wobble base pairing interactions (B). Colours in B) indicate base pairs that occur in essential wobble-pairing cognate tRNAs (red), and non-essential base pairs that were excluded in the analyses in figures 4 and 5 in the main manuscript.

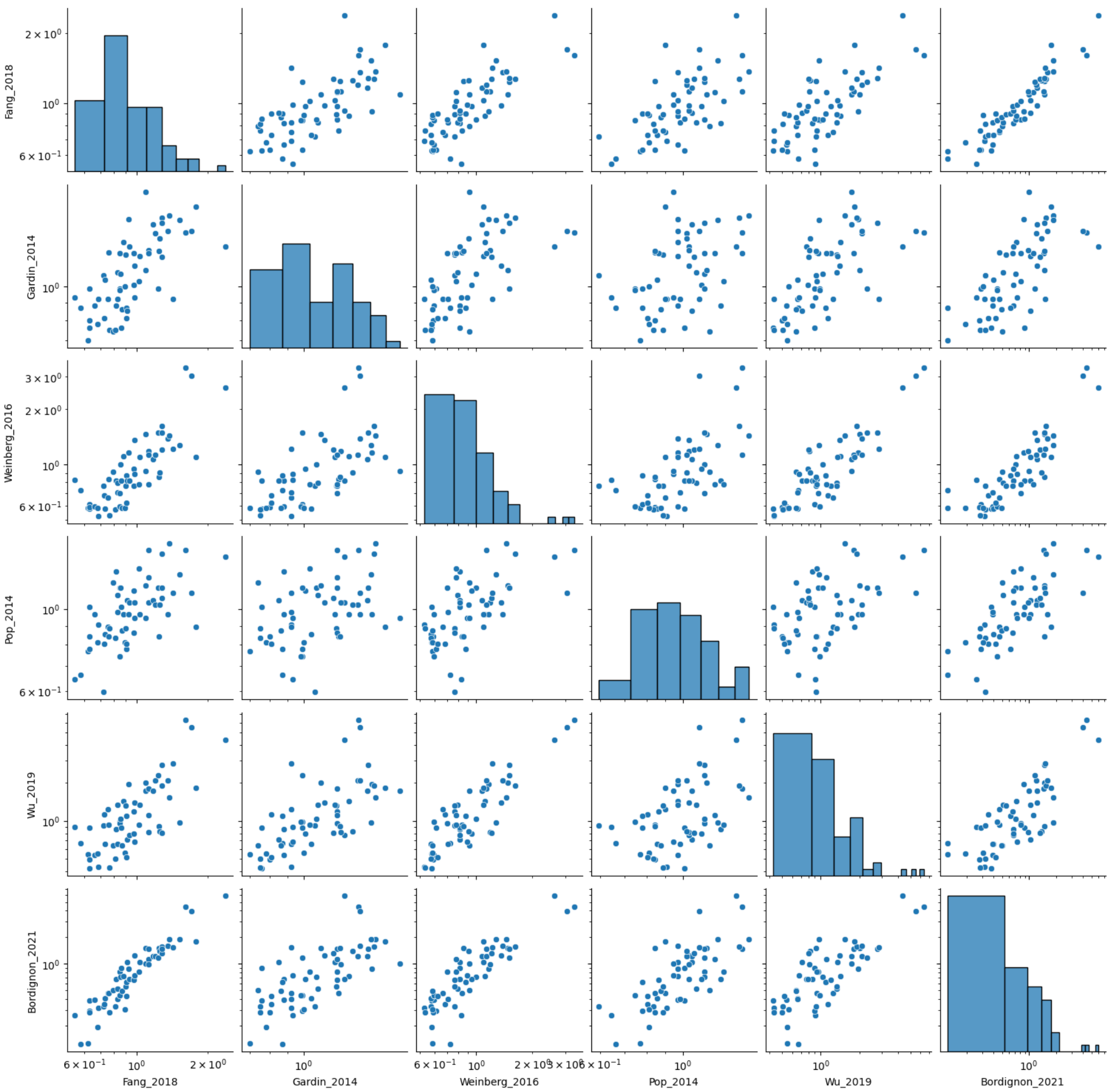

Supplemental figure 6. Pairwise comparison of published Ribosome Residence Time (RRT) datasets included in this study.

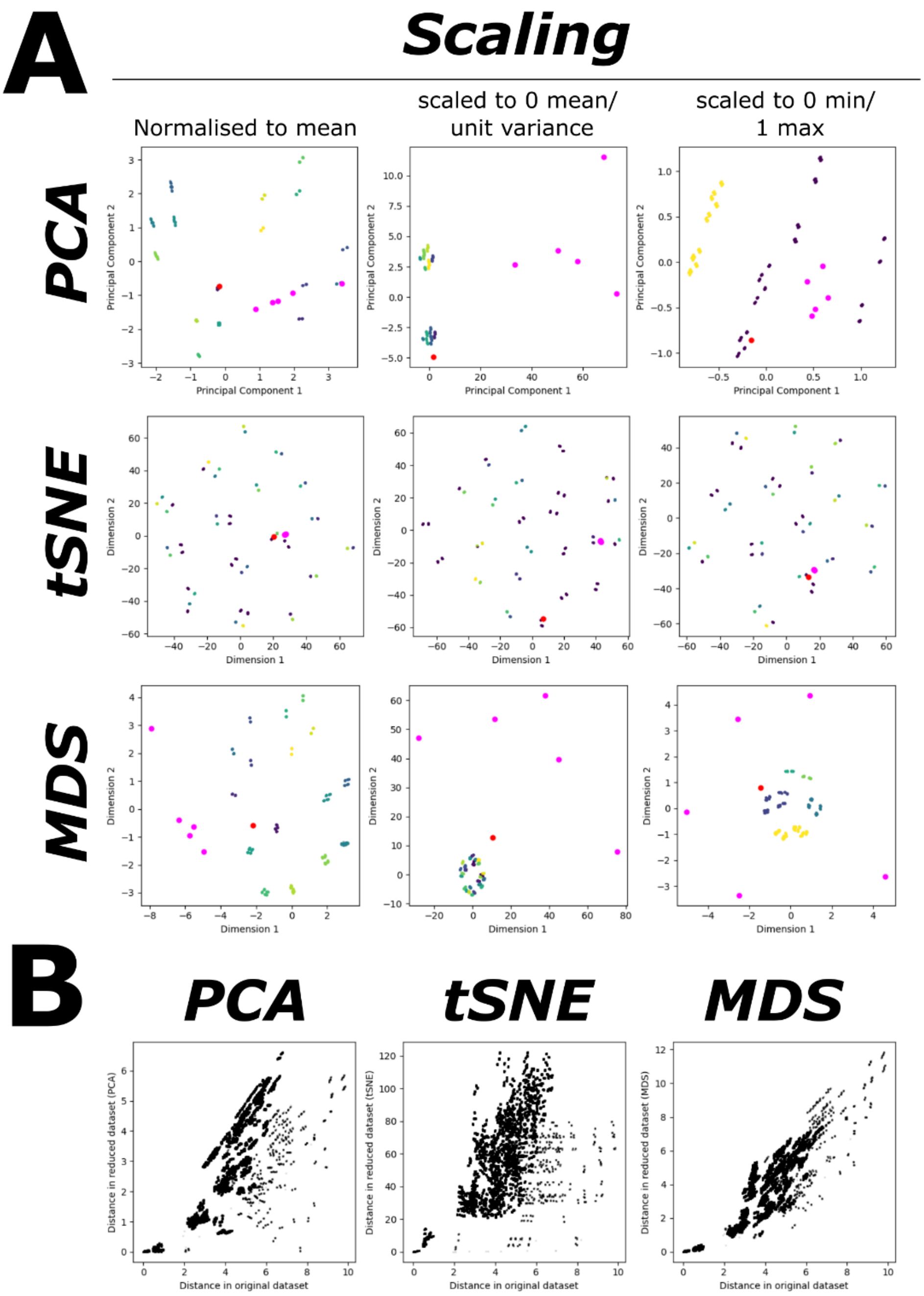

Supplemental Figure 7. Dimension reduction approaches applied to modelled codon decoding times. A, Principal Component Analysis (PCA), t-distributed Stochastic Neighbour Embedding (tSNE), and multi-dimensional scaling (MDS) were used to map Euclidean distances between modelled and experimental datasets, using different scaling approaches to normalise data. In all graphs, magenta data points represent experimental ribosome dwell time datasets; the red datapoint represents predicted codon decoding times with the full set of allowed nucleotide interactions, and all other datapoints represent modelled decoding times where one or more interactions were disallowed. Colours other than magenta and red identify datapoint clusters. Analyses in the main text refer to the MDS projection, for data which were normalised to the dataset mean. **B,** A comparison of Euclidean distances between the original datasets and between corresponding points in the different projections, for normalised to mean datasets.

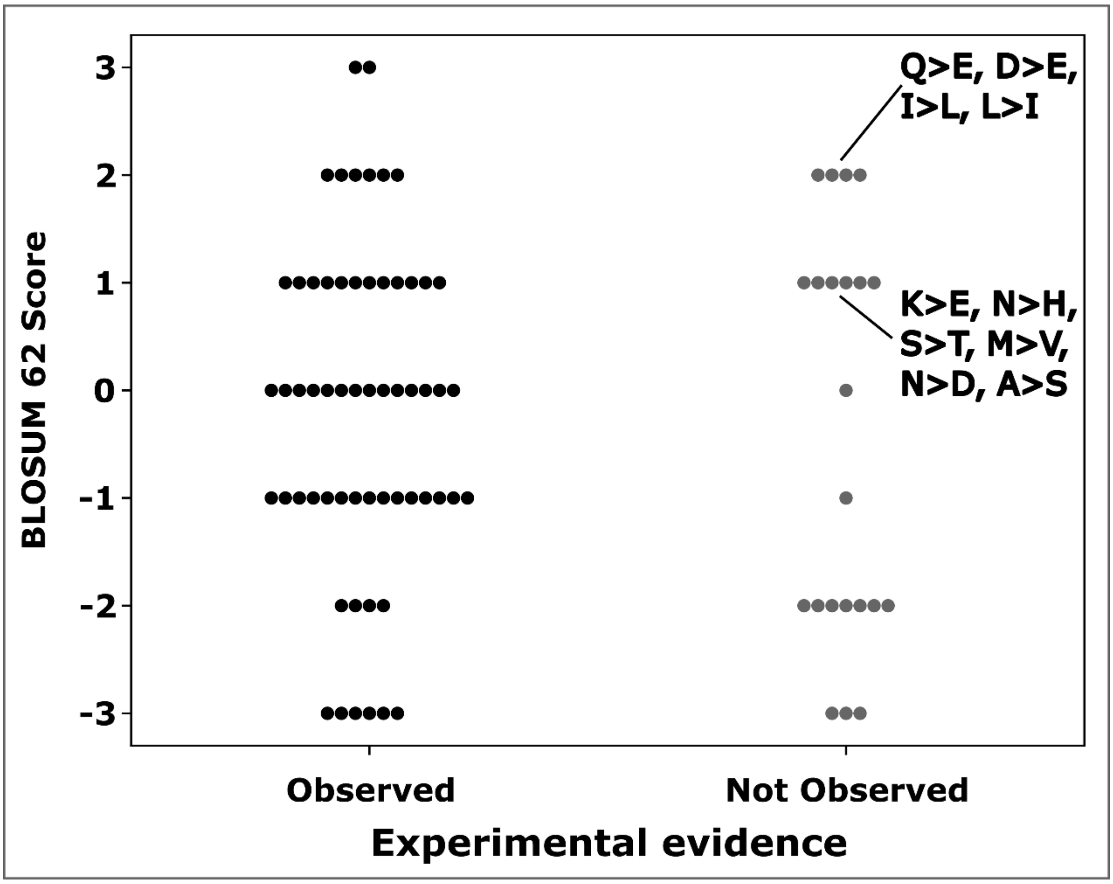

Supplemental Figure 8. Correspondence between amino acid substitution predicted by the near-cognate scheme used in simulations and experimentally observed substitutions. The majority of predicted substitutions 59 out of 81 predicted substitutions) have been experimentally observed in mass spectrometry-based assays (Landerer *et al.* 2024). The subset of predicted substitutions which have not been observed experimentally include substitutions not observable by mas spectrometry (I>L and L>I), and are enriched for substitutions with low BLOSUM scores indicating that these substitutions may result in unstable proteins, potentially limiting their detectability by mass spectrometry-based methods.

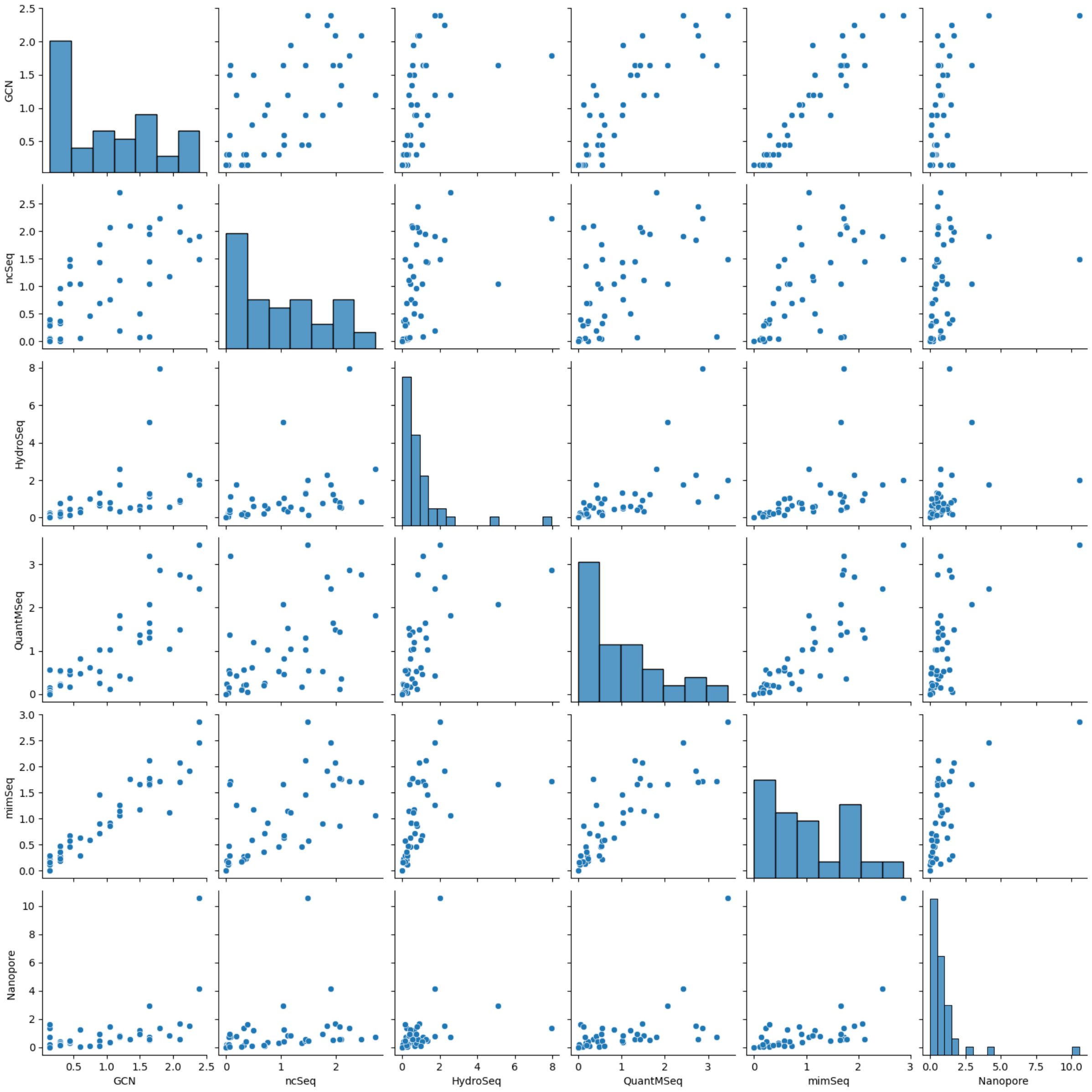

Supplemental figure 9. Pairwise comparison of published yeast tRNA datasets. Datasets are from NCBI GEO (ncSeq: GSE197651, HydroSeq: GSE109527, QuantMSeq: GSE198271, mimSeq: GSE152621), or from supplemental material of publications (Nanopore: Lucas *et al.* 2023). 'GCN' estimates tRNA levels based on the known gene copy number for each tRNA. Axes show relative abundance normalised to the mean for each dataset.

Origin state	Dest. state	tRNA species	Reaction name	Rate constant value	Source
0	1	non cognate	non1f	conc. dep.	
1	0	non cognate	non1r	2000 sec^{-1}	35,36
0	2	W:C cognate	WC1f	conc. dep.	
2	0	W:C cognate	WC1r	85 sec^{-1}	33
2	3	W:C cognate	WC2f	180 sec^{-1}	31,33,34
3	2	W:C cognate	WC2r	0.22 sec^{-1}	33,34
3	4	W:C cognate	WC3f	260 sec^{-1}	33
4	5	W:C cognate	WC4f	1000 sec^{-1}	[a]
5	6	W:C cognate	WC5f	1000 sec^{-1}	[a]
6	21	W:C cognate	WC6f	1000 sec^{-1}	[a]
6	0	W:C cognate	WCdiss	60 sec^{-1}	24,33
0	9	wobble cognate	wobble1f	conc. dep.	
9	0	wobble cognate	wobble1r	85 sec^{-1}	33
9	10	wobble cognate	wobble2f	190 sec^{-1}	33
10	9	wobble cognate	wobble2r	1 sec^{-1}	31
10	11	wobble cognate	wobble3f	25 sec^{-1}	31
11	12	wobble cognate	wobble4f	1000 sec^{-1}	[a]
12	13	wobble cognate	wobble5f	1000 sec^{-1}	[a]
13	21	wobble cognate	wobble6f	6.4 sec^{-1}	31
13	0	wobble cognate	wobblediss	1.1 sec^{-1}	31
0	16	near cognate	near1f	conc. dep.	
16	0	near cognate	near1r	85 sec^{-1}	33
16	17	near cognate	near2f	190 sec^{-1}	33
17	16	near cognate	near2r	80 sec^{-1}	33
17	18	near cognate	near3f	0.4 sec^{-1}	33
18	19	near cognate	near4f	1000 sec^{-1}	[a]
19	20	near cognate	near5f	1000 sec^{-1}	[a]
20	21	near cognate	near6f	60 sec^{-1}	24,33
20	0	near cognate	neardiss	1000 sec^{-1}	24,33
21	22	all	dec7f	200 sec^{-1}	24
22	23	all	trans1f	2040 sec^{-1}	24
23	22	all	trans1r	140 sec^{-1}	24
23	24	all	trans2	250 sec^{-1}	24
24	25	all	trans3	350 sec^{-1}	24
25	26	all	trans4	1000 sec^{-1}	[a]
26	27	all	trans5	1000 sec^{-1}	[a]
27	28	all	trans6	1000 sec^{-1}	[a]
28	29	all	trans7	1000 sec^{-1}	[a]
29	30	all	trans8	1000 sec^{-1}	[a]
30	0	all	trans9	1000 sec^{-1}	[a]

Supplemental table 1. Rate parameters names and value. The states correspond to states identified in figure 1 in the main text. Rate parameters designated as “conc. dep.” are calculated from tRNA abundance information for individual codons, using a second order rate of 140 μM^{-1} sec^{-1} (ref 31). All other rate parameters can be set via the “get_propensities()” / “set_propensities()” functions of the simulator. Default values which apply if no other values are specified are shown. For rate parameters where the source is given as [a], exact rates are not known but these steps are known to occur much faster than the rate limiting steps. For these rates a generic value of 1000 sec^{-1} was used.

tRNA Anticodon	relative signal						
	GCN	mimSeq	QuantMSeq	Nanopore	ARMSeq	HydroSeq	ncSeq
GAA	1.64	1.71	3.19	0.74	1.19	1.11	0.08
TAC	0.45	0.47	0.18	0.28	0.10	0.44	1.37
CAA	1.49	1.17	1.20	1.17	0.73	0.63	0.50
TAA	1.05	0.92	1.03	0.34	0.26	0.48	0.76
TCT	1.79	1.72	2.86	1.34	0.92	7.95	2.24
TTC	2.24	1.91	2.71	1.49	1.15	2.27	1.83
TTG	1.35	1.76	0.35	0.55	2.94	0.53	2.09
TTT	1.20	1.05	1.81	0.74	0.17	2.59	2.71
AGG	0.30	0.20	0.23	0.20	0.08	0.06	0.01
CCA	0.90	1.46	1.02	0.47	0.28	1.33	1.44
CAC	0.30	0.27	0.21	0.17	0.43	0.19	0.32
CAT	0.90	0.72	0.25	0.11	0.21	0.67	0.69
CCC	0.30	0.24	0.23	0.42	0.18	0.10	0.36
CCG	0.15	0.14	0.16	0.73	0.11	0.19	0.05
CCT	0.15	0.22	0.56	1.37	0.51	0.24	0.32
CGA	0.15	0.11	0.03	0.02	0.40	0.27	0.03
CGT	0.15	0.18	0.11	0.04	0.03	0.17	0.28
CTC	0.30	0.46	0.53	0.32	0.17	0.75	0.95
CTG	0.15	0.29	0.05	1.59	0.35	0.17	0.39
CTT	2.09	1.70	2.76	0.54	0.62	0.84	2.45
GAG	0.15	0.17	0.03	0.17	0.02	0.05	0.04
GAT	0.15	0.00	0.00	0.00	0.00	0.00	0.00
GCA	0.60	0.63	0.83	1.22	2.77	0.45	1.04
GCC	2.39	2.86	3.45	10.56	10.18	2.01	1.48
GCT	0.30	0.47	0.54	0.19	0.22	0.31	0.05
GTA	1.20	1.14	1.52	0.81	0.77	0.34	1.11
GTC	2.39	2.46	2.43	4.16	3.72	1.75	1.90
GTG	1.20	1.26	0.42	0.75	1.28	1.76	0.19
GTT	1.64	1.64	1.65	0.49	0.13	1.23	1.94
AAC	2.09	2.08	1.49	1.65	1.50	0.91	1.98
AAT	1.94	1.12	1.04	0.84	0.34	0.59	1.17
ACG	1.05	0.86	0.13	1.44	0.38	0.81	2.07
AGA	1.64	1.66	2.07	2.95	0.43	5.10	1.04
AGC	1.64	2.12	1.30	0.56	1.73	1.28	1.45
AGT	1.64	1.77	1.43	0.58	0.82	0.57	2.07
TCC	0.45	0.68	0.45	0.43	0.12	1.05	1.04
TGA	0.60	0.29	0.49	0.03	0.56	0.28	0.06
TGG	1.49	1.66	1.37	0.91	0.58	0.40	0.07
TAT	0.30	0.36	0.20	0.15	0.08	0.22	0.69
TAG	0.45	0.57	0.55	0.47	4.12	0.14	1.49
TGC	0.90	0.90	0.53	0.93	0.19	0.76	1.75
TGT	0.75	0.58	0.61	0.08	1.24	1.01	0.46

Supplemental table 2. tRNA abundance data generated with different methods. Data are plotted in in figure 5A in the main manuscript.

	tRNA dataset						
codon	HydroSeq	mimSeq	ncSeq	QuantMSeq	ARMSeq	GCN	Nanopore
AAA	0.18	0.55	0.26	0.36	2.20	0.51	0.43
AAC	0.44	0.34	0.32	0.39	3.31	0.36	0.99
AAG	0.29	0.29	0.25	0.21	0.57	0.25	0.45
AAT	0.67	0.57	0.54	0.63	3.75	0.59	1.28
ACA	0.47	0.40	0.34	0.40	0.26	0.37	0.86
ACC	0.61	0.42	0.37	0.41	0.37	0.40	0.80
ACG	0.65	0.69	0.65	0.72	0.41	0.65	2.97
ACT	0.51	0.25	0.20	0.27	0.28	0.25	0.58
AGA	0.07	0.21	0.22	0.13	0.38	0.18	0.31
AGC	2.59	1.14	9.66	1.10	4.02	1.56	4.94
AGG	2.76	1.57	1.17	0.75	0.68	1.99	0.27
AGT	2.97	1.45	10.78	1.41	4.52	1.92	5.41
ATA	0.60	0.55	0.43	0.66	1.58	0.46	0.58
ATC	0.81	0.75	0.64	0.78	1.93	0.50	0.69
ATG	0.46	0.67	0.68	1.99	2.28	0.62	2.72
ATT	0.58	0.50	0.40	0.55	1.60	0.31	0.45
CAA	1.14	0.37	0.36	1.95	0.14	0.51	0.85
CAC	0.32	0.56	4.00	1.65	0.49	0.57	0.83
CAG	1.14	0.52	0.51	2.13	0.31	0.69	0.26
CAT	0.54	0.81	4.46	1.98	0.72	0.83	1.08
CCA	1.41	0.34	5.60	0.34	0.64	0.35	0.41
CCC	9.40	3.42	28.14	2.67	5.51	2.34	2.51
CCG	8.70	2.82	26.26	2.29	4.29	1.97	2.33
CCT	8.80	2.98	26.84	2.25	4.91	1.96	2.13
CGA	1.24	0.71	0.46	3.90	1.25	0.57	0.54
CGC	1.31	0.89	0.47	5.13	2.87	0.69	0.99
CGG	3.64	2.82	7.96	2.68	3.32	2.37	0.65
CGT	1.02	0.62	0.25	4.62	2.51	0.45	0.72
CTA	2.56	1.03	0.36	1.07	0.11	1.49	0.84
CTC	1.99	0.96	0.52	1.26	0.31	1.29	0.78
CTG	2.61	1.14	0.52	1.21	0.30	1.58	0.94
CTT	2.09	1.03	0.52	1.30	0.32	1.38	0.85
GAA	0.27	0.38	0.49	0.29	0.53	0.33	0.38
GAC	0.40	0.28	0.41	0.34	0.12	0.30	0.12
GAG	0.35	0.49	0.46	0.43	0.67	0.49	0.54
GAT	0.63	0.50	0.65	0.56	0.32	0.53	0.33
GCA	0.43	0.32	0.21	0.42	0.39	0.34	0.34
GCC	0.50	0.36	0.33	0.44	0.39	0.39	0.45
GCG	1.04	0.80	0.47	1.12	2.40	0.82	0.62
GCT	0.35	0.20	0.23	0.29	0.20	0.25	0.36
GGA	0.83	0.85	0.60	1.41	7.11	1.14	2.43
GGC	0.42	0.19	0.42	0.17	0.07	0.20	0.08
GGG	7.52	2.24	1.41	2.80	4.77	1.56	2.37
GGT	0.66	0.40	0.65	0.38	0.27	0.41	0.28
GTA	0.40	0.39	0.27	0.55	0.56	0.43	0.37
GTC	0.63	0.50	0.50	0.63	0.59	0.54	0.45
GTG	0.76	0.97	0.50	1.67	1.15	1.06	1.07
GTT	0.40	0.27	0.28	0.40	0.37	0.31	0.23
TAC	1.73	0.62	0.69	0.44	0.82	0.59	0.76
TAT	2.11	0.85	0.96	0.68	1.08	0.83	1.04
TCA	0.28	0.48	0.65	0.36	0.49	0.40	0.32
TCC	0.31	0.57	0.69	0.46	1.21	0.57	0.33
TCG	2.02	4.08	9.99	10.46	0.77	3.30	15.15
TCT	0.10	0.35	0.44	0.24	0.93	0.35	0.12
TGC	1.87	0.86	0.52	0.72	0.32	0.79	0.84
TGG	0.53	0.25	0.32	0.40	1.32	0.39	1.01
TGT	2.21	1.13	0.77	0.98	0.54	1.09	1.13
TTA	0.76	0.63	0.74	0.56	2.12	0.62	1.19
TTC	0.29	0.32	5.39	0.16	0.45	0.38	0.51
TTG	0.36	0.31	0.49	0.31	0.55	0.29	0.23
TTT	0.52	0.54	5.82	0.37	0.69	0.61	0.76

Supplemental table 3. Predicted codon decoding times for different tRNA datasets. Data are plotted in figure 5B in the main manuscript.
